# Interspecific competition prevents the proliferation of social cheaters in an unstructured environment

**DOI:** 10.1093/ismejo/wrad038

**Published:** 2024-01-10

**Authors:** Hui Lin, Donglin Wang, Qiaojuan Wang, Jie Mao, Yaohui Bai, Jiuhui Qu

**Affiliations:** Key Laboratory of Drinking Water Science and Technology, Research Center for Eco-Environmental Sciences, Chinese Academy of Sciences, Beijing, 100085, China; University of Chinese Academy of Science, Beijing, 100049, China; Key Laboratory of Drinking Water Science and Technology, Research Center for Eco-Environmental Sciences, Chinese Academy of Sciences, Beijing, 100085, China; Key Laboratory of Drinking Water Science and Technology, Research Center for Eco-Environmental Sciences, Chinese Academy of Sciences, Beijing, 100085, China; University of Chinese Academy of Science, Beijing, 100049, China; Key Laboratory of Drinking Water Science and Technology, Research Center for Eco-Environmental Sciences, Chinese Academy of Sciences, Beijing, 100085, China; Key Laboratory of Drinking Water Science and Technology, Research Center for Eco-Environmental Sciences, Chinese Academy of Sciences, Beijing, 100085, China; Key Laboratory of Drinking Water Science and Technology, Research Center for Eco-Environmental Sciences, Chinese Academy of Sciences, Beijing, 100085, China

**Keywords:** kin bacteria, antibiotic impact, interspecific dynamics, social cheating, community coexistence

## Abstract

Bacterial communities are intricate ecosystems in which various members interact, compete for resources, and influence each other’s growth. Antibiotics intensify this complexity, posing challenges in maintaining biodiversity. In this study, we delved into the behavior of kin bacterial communities when subjected to antibiotic perturbations, with a particular focus on how interspecific interactions shape these responses. We hypothesized that social cheating—where resistant strains shield both themselves and neighboring cheaters—obstructed coexistence, especially when kin bacteria exhibited varied growth rates and antibiotic sensitivities. To explore potential pathways to coexistence, we incorporated a third bacterial member, anticipating a shift in the dynamics of community coexistence. Simulations and experimental bacterial communities confirmed our predictions, emphasizing the pivotal role of interspecific competition in promoting coexistence under antibiotic interference. These insights are crucial for understanding bacterial ecosystem stability, interpreting drug–microbiome interactions, and predicting bacterial community adaptations to environmental changes.

## Introduction

Most habitats on earth are populated by diverse bacterial communities [1–3]. Within these complex assemblages, individual members engage in signaling [4, 5] and resource competition [6–8] as well as the active production of substances that can benefit or harm others within the community [9, 10]. Unraveling the mechanisms that maintain bacterial community biodiversity poses a considerable challenge for evolutionary ecology, as the competitive nature of interactions often results in the exclusion of competitively inferior community members, especially when communities are exposed to antibiotics. Such exposure introduces intricate social dilemmas, marked by the emergence of “cooperators”—those facilitating group benefits—and “cheaters” who exploit these benefits, altering the community structure [11, 12]. To address these dilemmas and promote coexistence, several mechanisms, such as partial privatization and spatial structuring, have been proposed [13–15]. For instance, using individual-based modeling, a recent study suggested that bacteria have evolved coexistence maintenance mechanisms, which enforced cooperation through the costly synthesis of a harmful toxin that selectively targeted noncooperative cheaters [16]. Additionally, in toxin-mediated interference competition, spatial structure can support coexistence by offering weaker members sanctuaries from their more competitive counterparts [17, 18]. These dynamics can give rise to cyclic dominance among species (e.g. “rock–paper–scissors” game), beyond the constraints imposed by competitive exclusion [19, 20].

Much of these existing studies centered on addressing social dilemmas that arise among different bacterial species when faced with antibiotic interference [21–25]. Nevertheless, empirical evidence from natural ecosystems suggested that different genotypes (strains) of the same bacterial species also engaged in a broad spectrum of interactions [4, 26–28]. These intraspecific interactions ranged from intense competitions, often due to vying for the same nutrients and space, to cheating and altruistic behaviors like the secretion of public goods such as extracellular enzymes or metabolites that benefit the entire community [28–32]. Moreover, kin bacteria frequently thrived in well-mixed, spatially unstructured environments, making cooperators easier for cheaters to exploit [33, 34]. The intricacies of how these kin bacteria coexist under the perturbation of antibiotics remain largely unexplored.

Here, we explored how increased interspecific interactions influenced the coexistence of kin bacteria within unstructured and well-mixed habitats in the presence of temporary antibiotic perturbations. We postulated that a prevalent form of intraspecific interaction among kin bacteria manifested as social cheating under antibiotic stress. In such circumstances, resistant strains effectively removed antibiotics from nearby locations, thereby protecting themselves and neighboring cheaters of the same species. Coexistence became challenging when there were significant differences in the inherent growth rates and competitive abilities between antibiotic-resistant (cooperator) and -sensitive (cheater) strains. By employing the *Comamonas testosteroni* KF-1 (cooperator)–CNB-2/Δ*LuxR* (cheater) experimental community exposed to sulfamethoxazole (SMX) perturbation, we validated these predictions. While consistent with the competitive exclusion principle, introducing a third member—termed the “regulator”—into the community was hypothesized to foster balance between cooperator and cheater strains. However, this addition could intensify inhibitory interactions, due to the potential production of multiple antibacterial agents [35, 36]. Hence, we expanded our theoretical model to simulate this three-way interaction, identifying feasible conditions for coexistence between these kin bacteria in well-mixed environments. Our results demonstrated that this coexistence relied on three-way competitive interactions, rather than facilitation, with the involvement of an external species as a regulator. The emergence of interspecific competition mitigated intraspecific inhibitory interactions, allowing coexistence despite differences in the inherent growth rates and the exploitation of cooperators by cheaters. We validated our model by introducing *Pseudomonas aeruginosa* into the experimental community, revealing that interspecific competitive interactions exert a strong enough force to foster kin bacteria coexistence and biodiversity.

## Materials and methods

### Bacterial strains

Two *C. testosteroni* strains were used in the study, *C. testosteroni* KF-1 (SMX-resistant, accession no. NBRC 100989) and *C. testosteroni* CNB-2/∆*LuxR* (SMX-sensitive, ATCC 11996). KF-1, served as the cooperating strain, can produce *LuxR solo* protein that binds to free-SMX and stimulates its cell growth [37]. In contrast, CNB-2/∆*LuxR* acted as the cheating strain, lacking *LuxR solo* protein and thereby unable to resist SMX, leading to the manifestation of an inhibitory effect [37]. Synthetic communities consisting of these two strains were used to investigate intraspecific cheating behavior. Then, *P. aeruginosa* PAO1 (ATCC 15692) was chosen as the regulatory species due to its growth rate falling between the two *C. testosteroni* strains. We examined three distinct phenotypes in response to SMX: SMX-sensitive (PAO1), hormetic-resistant (PAO1-*LuxR*), and intrinsic-resistant (PAO1-*Sul1*). PAO1-*LuxR* was engineered to express the *LuxR solo* gene via pFPV-*LuxR* plasmid transduction, enabling it to possess the ability to bind free-SMX. Furthermore, PAO1-*Sul1* carried the classical SMX resistance gene *Sul1* through pFPV-*Sul1* plasmid transduction, providing intrinsic resistance to SMX. All bacterial strains, gene deletion mutants, and plasmids used in this study are listed in Table S1. All primer pairs used for gene complementation are listed in Table S2.

### Bacterial interaction assays

Overnight liquid precultures of all strains were grown separately in nutrient broth (NB) medium at 30°C/200 rpm. To assess the competitive relationship between the KF-1 and CNB-2/Δ*LuxR* strains, we washed the overnight monocultures twice with phosphate-buffered saline (PBS) buffer. We then mixed the strains at 1:1 ratios and inoculated the mixtures at a 3% inoculation proportion (v:v) into 50 ml of mineral salt medium: 10 mM C_2_H_3_O_2_Na, 4 mM KNO_3_, 2 mM MgSO_4_•7H_2_O, 0.4 mM CaCl_2_•2H_2_O, 4 mM KH_2_PO_4_, 10 mM HEPES, 0.001 mg/l vitamin B_1_, 0.002 mg/l vitamin B_12_, and 0.003 mg/l biotin. At the end of each daily cycle, we diluted the experimental cultures 10-fold by transferring 5 ml of the culture into 50 ml of fresh MSM. For the experiments involving community exposure to antibiotics, we supplemented the MSM with 200 μg/l SMX before daily dilution on Day 3. For the coexistence experiments involving three members, we supplemented the MSM with 5 mM glucose.

### Estimation of population densities

To quantify the colony-forming units (CFU) of the *C. testosteroni*, we used NB-agar plates supplemented with either SMX (10 μg/ml, Sigma cat# S7507) or gentamycin (30 μg/ml, Sigma cat# E003632) to select for the KF-1 and CNB-2/Δ*LuxR*, respectively (Table S3). To differentiate the *P. aeruginosa* colonies, we used *Pseudomonas* CN–agar plates (Hopebio cat# HB8484-2) and identified them based on their distinct green color (Table S3). The traditional method of CFU counting through serial dilution and plating with glass beads on agar plates was used. 10 μl of PBS-serial diluted cultures (dilution factor ~10^5^ to ~10^9^) were plated on 90-mm-diameter agar plates with or without antibiotic selection.

### Measurement of growth rates

To assess the growth rates, we prepared monocultures with an initial population of 10^9^ cells for all strains. Subsequently, a gradient dilution step was performed to obtain monocultures spanning a range of initial cell densities (~10^5^ to 10^9^). These monocultures were then incubated in 96-well MSM plates, both with and without SMX, with vigorous continuous shaking at 30°C. The plates were tightly sealed, and OD was monitored using a Spark 10-M microplate reader (Tecan) at a wavelength of 600 nm (OD_600_) for 72 hours. Growth rates were measured by assuming exponential growth to a threshold of OD 0.5, and the effective growth rate was determined by calculating the ratio lg(OD_threshold_/OD_i_)/T_threshold_, where T_threshold_ represents the time taken by each population to reach an OD threshold (OD_threshold_ = 0.5 cm^−1^), and OD_i_ represents the initial OD of that particular population.

### Simulations

In our numerical simulations, we utilized the ode() function from the deSolve package in R. All parameters utilized in simulations were derived from our experimental system, including growth rates, death rates, and disturbance times. Key parameter values were:


$$ {\beta}_{max}=0.5\ \left[{h}^{-1}\right] $$

$$ {\alpha}_{max}=0.75\ \left[{h}^{-1}\right] $$

$$ {\alpha}_{BA}=1.75 $$

$$ {\alpha}_{AB}=1.9 $$

$$ {a}_A={a}_B=0 $$

In our simulations, death rates applied only during 72–96 hours, with zero rates afterward; under antibiotic conditions, Member A had a death rate of μ_A_ = −0.6, while Member C’s rates varied: μ_C_ = 0.8 for sensitive, μ_C_ = −0.3 for detoxifying, and μ_C_ = 0 for intrinsically resistant cells. Each simulation had a total runtime of 168 hours. To generate our phase diagrams, we employed a step size of 500, providing detailed insights into the community dynamics. When varying growth rate β_B_ in Fig. 3B, we held α_A_ = 0.5 constant while reducing β_B_. Moreover, for the death rate in Fig. S3, we retained μ_A_ = −0.6 as a constant and adjusted the initial cell density D.

## Results

### Construction of the experimental system

**Figure 1:**
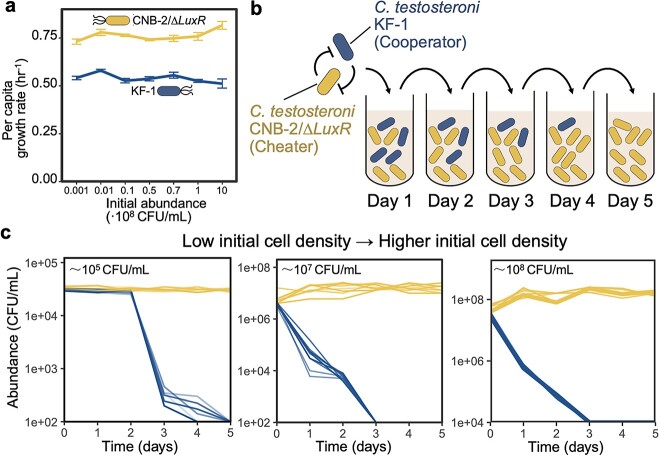
Growth rate determined intraspecific interaction outcomes in environments free from antibiotic interference; (A) *C. testosteroni* CNB-2/∆*LuxR* exhibited a higher growth rate than KF-1 across inoculum densities of 10^5^ to 10^9^ CFU/ml; effective growth rates of monocultures inoculated at different initial cell densities (mean ± SD, *n* = 6) were shown; the per capita growth rate referred to the population growth rate normalized by the initial population size; (B) co-culture of CNB-2/∆*LuxR* and KF-1 was diluted by a factor of 10 each day (1/10 of previous day’s culture transferred to fresh medium, with constant volume); (C) fast-growing CNB-2/∆*LuxR* dominated and drove slow-growing KF-1 to extinction (*n* = 9).

**Figure 2:**
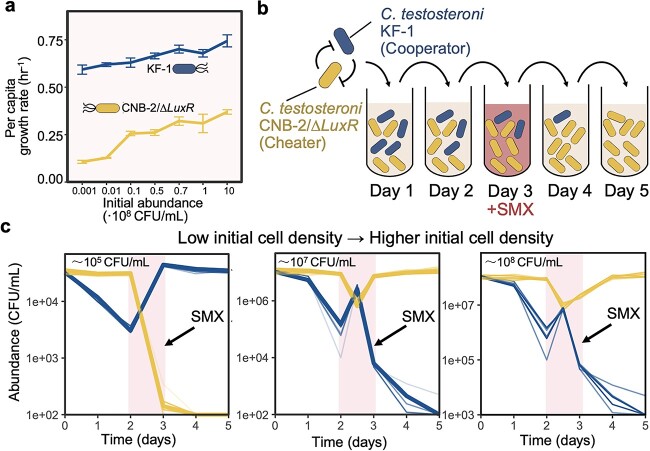
Sulfamethoxazole (SMX) exposure triggered intraspecific social cheating; (A) under 200 μg/l SMX exposure, KF-1 showed a higher growth rate than CNB-2/∆*LuxR* across inoculum densities of 10^5^–10^9^ CFU/ml; effective growth rates of monocultures at different initial cell densities (mean ± SD, *n* = 6) were shown; the per capita growth rate referred to the population growth rate normalized by the initial population size; (B) co-culture of CNB-2/∆*LuxR* and KF-1 was performed using serial dilution, revealing two stable states; (C) within these states, cooperator KF-1 dominated the community at low initial cell density, while cheater CNB-2/∆*LuxR* dominated at higher initial cell density; pink highlights 24-hour cycle under SMX exposure (*n* = 9).

We began by establishing a model community comprised of two *C. testosteroni* variants: KF-1 and CNB-2/Δ*LuxR*. These strains are prevalent in diverse environments, including activated sludge, marshes, marine habitats, and animals, plants, and human tissues [38, 39]. Their broad adaptability positions them as potential agents of mild yet persistent infections, an issue that is garnering increased attention [40, 41]. These strains have been identified as causative agents for a range of conditions, including cellulitis, peritonitis, endocarditis, meningitis, endophthalmitis, tenosynovitis, pneumonia, and bacteremia [42]. Even though KF-1 and CNB-2/Δ*LuxR* belong to the same species, they manifest distinct growth characteristics and sensitivities when exposed to the antibiotic SMX. The KF-1 variant, although a weaker competitor with a slower inherent growth rate, grew better in SMX environments due to its ability to produce *LuxR* solo proteins that effectively bind to free SMX [37]. In contrast, CNB-2/Δ*LuxR* exhibited a significantly elevated growth rate (Wilcox-test, *P* < .05 across all initial cell densities) but was susceptible to SMX because of the absence of *LuxR* solo after genetic modification (Figs 1A and 2A). In the present study, CNB-2/Δ*LuxR* and KF-1 provided an ideal experimental model for studying shifts in stable states and social cheating behaviors following antibiotic interference. Given the considerable overlap in their ecological niches and contrasting growth characteristics, we anticipated mutual inhibitory interactions between these strains.

### Shifts in stable states and social cheating behavior after sulfamethoxazole exposure

In a nutrient-limited mixed environment, we co-cultured two strains using a serial dilution protocol (Fig. 1B). This protocol mimicked a chemostat operating at a fixed dilution rate, which permitted bacteria to grow in one growth phase with a fixed doubling time and better control of the environment and cell density. In our observations, the CNB-2/∆*LuxR* strain swiftly overtook the KF-1 strain, resulting in its extinction within three growth cycles (Fig. 1C), underlining the anticipated mutual inhibition between them.

Given the insights from previous research emphasizing that pulse perturbations may have the ability to transition the microbiome from its customary equilibrium to a different state [43], we explored if transient SMX exposure could induce a shift in the steady-state dominance due to the varying antibiotic susceptibility of the strains. Recognizing the resistant strain KF-1’s ability to counteract SMX’s inhibitory effects through SMX binding, we also probed into the potential consequences of the inoculum density effect. This effect implied that higher inoculum densities of the resistant strain would lead to a more substantial reduction in inhibition effect, ultimately conferring benefits to the entire population [44]. We assessed synthetic communities subjected to a one-cycle SMX exposure, adjusting the inoculum densities from 10^5^ to 10^8^ CFU/ml (Day 3, Fig. 2B). Our observations highlighted that the addition of 200 μg/l SMX over a single day produced distinct shifts in community composition. When the initial inoculum density was low (~10^5^ CFU/ml), the sensitive CNB-2/∆*LuxR* strain exhibited susceptibility to SMX, leading to a pronounced dominance of the resistant KF-1 strain (Fig. 2C). However, higher inoculum densities led to a reversal, with CNB-2/∆*LuxR* becoming dominant (~10^7^ and 10^8^ CFU/ml, Fig. 2C). This shift indicated that higher levels of protection against SMX were attained by CNB-2/∆*LuxR* with increasing KF-1 density. By analyzing SMX concentration across varying initial cell densities, we found that KF-1 linearly reduced SMX levels through its binding action (Fig. S1A). This rate of SMX clearance intensified with increasing initial cell density (Fig. S1B). With the decline in SMX levels, the death rate of CNB-2/∆*LuxR* also showed a downward trend (Fig. S1C). As a result, when the initial cell densities were high enough, the rapid decline in antibiotic concentration led to a significant reduction in the average mortality of the sensitive CNB-2/∆*LuxR* strain, within the interference cycle (Fig. S1D). This dynamic effectively provided cross-protection for CNB-2/∆*LuxR*.

Furthermore, we investigated whether the community shift observed after SMX exposure was due to CNB-2/∆*LuxR* acquiring resistance through horizontal gene transfer. We isolated CNB-2/∆*LuxR* cells co-cultured with KF-1 after SMX exposure and examined their growth rates in a new medium containing SMX. Our findings revealed that the surviving cells displayed the same inhibitory response to SMX as the naive cells (Fig. S2), indicating that community shift was not the result of resistance acquisition. Hence, the strain CNB-2/∆*LuxR* acted as a cheater, exploiting the cross-protection behavior of the cooperator, KF-1.

### Modeling dominance of cheaters with higher growth rates in bacterial communities regardless of antibiotic susceptibility

**Figure 3:**
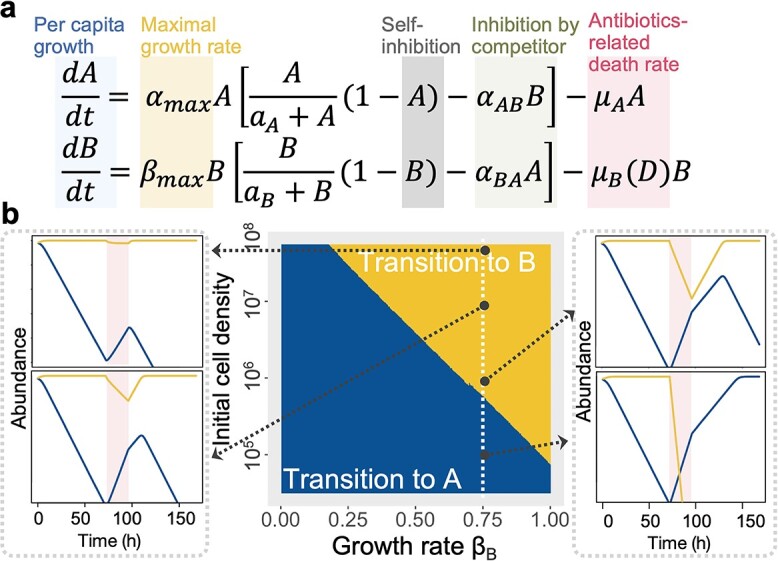
Understanding community dynamics using a modified Lotka–Volterra (LV) model; (A) we applied a refined LV competition model, accounting for the per capita growth rate, self-inhibition, initial cell density, intraspecific interaction, and antibiotic-induced death rate; (B) the phase diagram depicted the model’s predictions on how antibiotic exposure influences community dynamics; the horizontal axis represented the growth rate of the Cheater B, while the vertical axis indicated the initial cell density; representative time series further illustrated these dynamics over time; as the initial population increased, community shifts were evident: moving from a dominance of Cooperator A to a dominance of Cheater B.

We next sought to explore the prevalence of such dynamics and identify the factors that facilitate the success of a cheater in an antibiotic-exposed environment. We applied a refined Lotka–Volterra (LV) competition model, modified to include antibiotic-induced additional death (Fig. 3A):


$$ \frac{dA}{dt}={\alpha}_{max}A\left[\frac{A}{a_A+A}\left(1-A\right)-{\alpha}_{AB}B\right]-{\mu}_AA $$

$$ \frac{dB}{dt}={\beta}_{max}B\left[\frac{B}{a_B+B}\left(1-B\right)-{\alpha}_{BA}A\right]-{\mu}_B(D)B $$

In our model, we employed normalized abundances for the cooperator (A) and cheater (B) strains, designated as A and B, respectively. The parameters α_max_ and β_max_ represented the maximum per capita growth rates of the cooperator and cheater strain. The strength of intraspecific interaction was captured by α_AB_ and α_BA_. The parameters a_A_(a_B_) introduced an Allee effect, representing the strength of the density-dependent individual fitness for the respective strains. For strains that do not experience any influence of density on their growth, a_A_ and a_B_ were defined as zero, indicating the absence of any Allee effect for those particular strains. The parameters μ_A_ and μ_B_ signified the average death rates related to antibiotic exposure for each strain, reflecting their respective susceptibilities to transient antibiotic presence. Among them, μ_B_ was delineated as a linear function of the initial cell density, indicating that the survivability of cheater strains depended on the population of cooperators able to neutralize the antibiotic’s impact.

While our model neglected many aspects of bacterial growth in antibiotics, it successfully captured key dynamic features of cooperators and cheaters. Under antibiotic perturbation, the abundance of B, representing the cheater strain, declined due to its susceptibility to antibiotics (accounted for as an added death rate in the model). In contrast, the abundance of A, representing the cooperator strain, increased owing to its enhanced growth rate, driven by the hormetic effect (depicted as a negative death rate in the model). Our simulations showed that at low initial densities, the antibiotic hormetic/resistance of the cooperator allowed them to dominate the community (Fig. 3B, middle panel). This trend emerged regardless of variations in the intrinsic growth rates of cheaters, possibly due to the delayed protection for susceptible cells. This extended the time required for antibiotic concentrations to decrease below the minimum inhibitory concentration for the cheater. In contrast, when there was a larger initial population of the cooperator, the heightened degradation rates neutralized the antibiotic effects and the inherent differences in growth rates propelled the cheater to take the lead (Fig. S3). Moreover, using the experimentally determined growth rate of cheater [β_B_ = 0.75], our simulations corroborated the experimental observation that as the initial cell density increased, the community’s post-antibiotic exposure state shifted from being predominantly cooperator to predominantly cheater (Fig. 3B, left and right). The former scenario demonstrated that Cooperator A gained an absolute advantage during antibiotic exposure (Fig. 3B, right). In contrast, the latter scenario revealed that cooperative behavior was susceptible to exploitation by Cheater B that benefited from the assistance of cooperators (Fig. 3B, left). Therefore, this simple model effectively recapitulated the core experimental outcomes observed in our bacterial community exposed to different antibiotics. It demonstrated that the more susceptible strain could still dominate the community after antibiotic exposure in the presence of a detoxified strain.

### Modeling the response of a three-member community to antibiotics

Previous studies have highlighted the influence of additional competitors and network structures on species competition outcomes [45–48]. Building upon this, we proposed that coexistence could be facilitated when a third member selectively exerts pressure to attenuate intraspecific exploitation between the other two strains. We expanded our model beyond the two-member system to incorporate the presence of a regulatory member. Our goal was to identify key characteristics of regulator C that facilitate community coexistence by analyzing the behaviors of its three SMX phenotypes: antibiotic-sensitive, antibiotic-detoxification, and intrinsically resistant strains.


$$ \frac{dA}{dt}={\alpha}_{max}A\left[\frac{A}{a_A+A}\left(1-A\right)-{\alpha}_{AB}B-{\alpha}_{AC}C\right]-{\mu}_AA $$

$$ \frac{dB}{dt}={\beta}_{max}B\left[\frac{B}{a_B+B}\left(1-B\right)-{\alpha}_{BA}A-{\alpha}_{BC}C\right]-{\mu}_B(D)B $$

$$ \frac{dC}{dt}={\gamma}_{max}C\left[\frac{C}{a_C+C}\left(1-C\right)-{\alpha}_{CA}A-{\alpha}_{CB}B\right]-{\mu}_CC $$

The interactions between the members were described by α_ij_, representing the benefits or detriments that member i received from member j. Bidirectional interactions between the regulatory and native member (α_AC,_ α_BC_) were classified as competition (−/−), exploitation (−/+), or mutualism (+/+) [27] (Fig. S4A). We assumed a complete correlation between the interaction coefficients of any pair of members (γ ≡ corr(α_ij_,α_ji_) = 1, then α_ij_ = α_ji_).

In each simulation, we initially assembled three members at equal proportions that interacted according to competition coefficients. These communities grew from a fixed total initial cell density, and after a daily cycle, the culture was diluted back to the initial cell density. Consequently, members with higher growth rates within each round were over-represented in the subsequent round. This can be seen as competition for space in the inoculum for the next round, promoting the increase of members that, overall (due to their basal growth rate and interactions with other members), exhibited the fastest growth. We defined coexistence operationally as the persistence of members in the community after a given amount of community growth (in this case, a 5-day culture, 100 generations), which matched our experimental system and allowed us to balance experimental feasibility and capture community dynamics over biologically relevant time scales. This approach aligned with principles presented in prior studies on microbial community coexistence, emphasizing the dynamic balance within microbial communities over specific time frames [23–25]. We examined the population size of each member in the final cycle of community growth and compared it to the initial cell density. If any one member’s population size decreased by more than 20% in the final cycle, we classified the community as not coexisting, as it indicated a potential pathway toward extinction.

**Figure 4:**
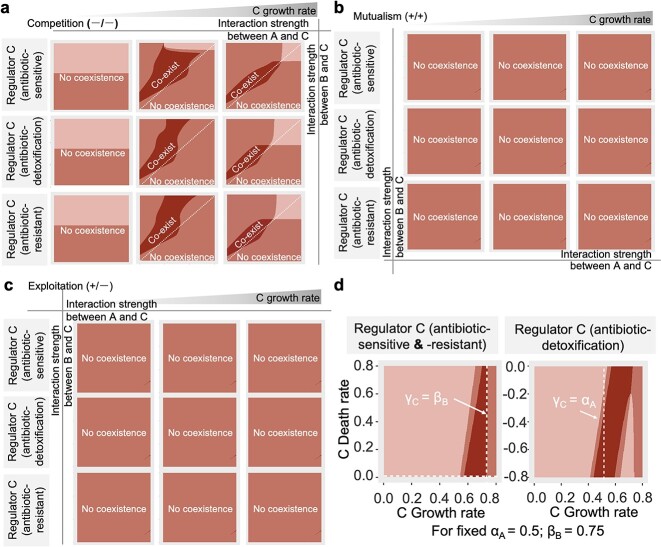
Regulator species enhanced species coexistence and biodiversity; phase diagram demonstrated profound influence of interaction strength (A–C) and growth rate (D) on community coexistence and dynamics; the simulation encompassed three categories of bidirectional interactions: competition (where both α_AC_ and α_BC_ were positive), mutualism (where both α_AC_ and α_BC_ were negative), and exploitation (where either α_AC_ or α_BC_ was positive).

Our simulations demonstrated that the coexistence and dynamics of the community heavily relied on both the growth rate and interaction strength (<α_ij_>) between members, regardless of their antibiotic sensitivity (Fig. 4). We observed that for the community to remain stable, all pairwise interactions between the three members needed to be negative, with a greater negative effect on the cheater compared to the cooperator (Fig. 4A–C and Fig. S4B and C). When the growth rate of the Regulator C increased under negative pairwise interactions, stable coexistence was achieved within a specific region (Fig. 4D and Fig. S4D). Here, Regulator C’s growth rate closely mirrored that of Cheater B and exhibited a moderate growth rate/mortality ratio (|γ_C_/μ_C_|). This exerted competitive pressure on the populations of both Cooperator A and Cheater B, fostering the coexistence of all three community members. Moreover, this equilibrium persisted and remained unchanged in the absence of additional disturbances (Fig. S5).

### Experimental validation of coexistence dynamics in a three-member bacterial community under sulfamethoxazole interference

**Figure 5:**
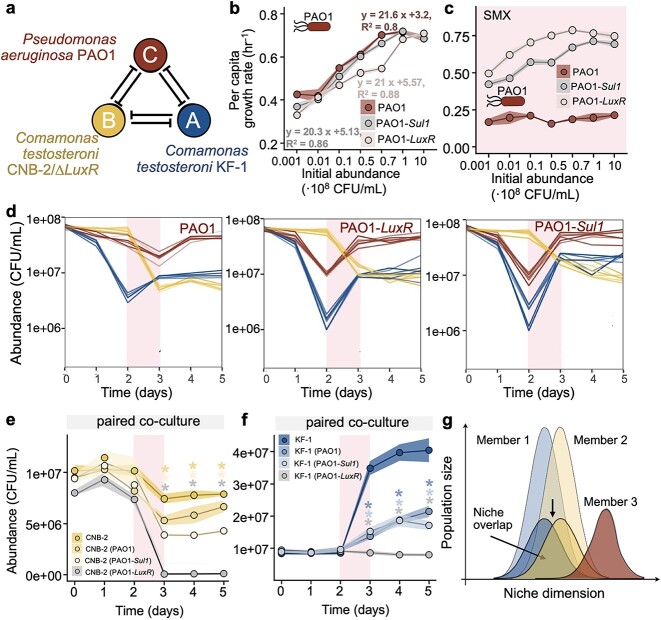
Coexistence dynamics of three members in a well-mixed environment during antibiotic perturbation; (A) three-way interactions among KF-1, CNB-2/∆*LuxR*, and *Pseudomonas aeruginosa*; growth rates of three sulfamethoxazole (SMX) response phenotypes of *P. aeruginosa* (PAO1, PAO1-*LuxR*, and PAO1-*Sul1*) were measured under two conditions: (B) absence and (C) presence of 200 μg/l of SMX; the per capita growth rate referred to the population growth rate normalized by the initial population size. Equation depicted the relationship, while R^2^ indicated the linear correlation between growth rate and initial cell density. In all regression analyses, the *t*-test yielded *P*-values <.05; (D) coexistence performance of a three-species community was evaluated using a serial dilution protocol; (E) and (F) showed the comparison of mono- and pairwise co-cultures; population size was quantified in CFUs/ml over time for monocultures and pairwise co-cultures; significance levels (^*^P < .05) were assessed using the Wilcox test each cycle (Table S6 and S7); (G) our hypotheses proposed that increased interspecific interactions play a crucial role in promoting species coexistence in unstructured, well-mixed habitats when bacterial species encountered antibiotic perturbations.

We conducted laboratory experiments to validate the findings of modeling regarding the coexistence dynamics of a three-member community. The synthetic community consisted of two *C. testosteroni* strains and one *P. aeruginosa* strain. To induce competitive pairwise interactions within the community, we designed resource–competitive media that contained two different carbon sources (sodium acetate and glucose) and a nitrogen source (potassium nitrate). Due to their inability to metabolize glucose [49–53], the two *C. testosteroni* strains relied solely on acetate as their carbon source. This specific trait led to a significant decline in cell densities when they were cultivated in a glucose-only medium (Fig. S6A and B). As a result, the coexistence dynamics of these two variants in an environment with both carbon sources (sodium acetate and glucose) was consistent with the dynamics observed in the environment of a single carbon source (sodium acetate) (Fig. S6C). Conversely, *P. aeruginosa* exhibited a broader metabolic capacity and utilized both acetate and glucose. All three members competed for the single available nitrogen source. The two *C. testosteroni* strains competed for both carbon and nitrogen resources, whereas *P. aeruginosa* primarily competed for nitrogen, with access to a relatively abundant supply of carbon. This experimental setup facilitated the establishment of a synthetic community characterized by competitive pairwise interactions, with *P. aeruginosa* possessing a significant resource advantage (Fig. 5A).

We initiated our analysis by examining the growth rate of the three members in monoculture conditions. As mentioned earlier, CNB-2/∆*LuxR* consistently exhibited a significantly higher growth rate than KF-1, regardless of the initial inoculum density. In contrast, in *P. aeruginosa*, while there were no significant differences in the growth rates of the three different SMX phenotypes, changes in growth rates with initial inoculum density were observed in all of them (Fig. 5B and Table S4). Consequently, *P. aeruginosa* displayed distinct growth rate rankings at different initial inoculum densities: at lower initial inoculum densities (<10^6^ CFU/ml), *P. aeruginosa* had lower growth rate rankings than the two *C. testosteroni* strains. At higher initial inoculum densities (>10^8^ CFU/ml), *P. aeruginosa*’s growth rates ranked higher than those of the two *C. testosteroni* strains. The medium inoculum density (~10^7^ CFU/ml) fell in between these two extremes (Table S4). Additionally, the PAO1-*Sul1* strain showed no significant change in growth rate with SMX (Wilcox test *P* = .0598), while PAO1-*LuxR* had a significant increase (Wilcox test *P* = 6e-05), and PAO1 was significantly inhibited by SMX (Wilcox test *P* = 8e-05), as expected (Fig. 5C and Table S5). Then, we tested whether the addition of *P. aeruginosa* resulted in the coexistence of the two *C. testosteroni* strains. We used the previous 5-day serial dilution protocol to assess the synthetic community dynamics. Our findings showed the establishment of a stable coexistence state for *C. testosteroni* strains alongside all *P. aeruginosa* phenotypes of medium growth rate ranking (~10^7^ CFU/ml) under SMX interference(Fig. 5D), and it persisted across multiple transfers. However, coexistence was absent at both lower (~10^5^ CFU/ml) and higher (~10^8^ CFU/ml) initial inoculum densities (Fig. S7A and B). We speculated that these absences were linked to shifts in the growth rate ranking of *P. aeruginosa*, which hindered coexistence in synthetic communities. Lower rankings inhibited successful *P. aeruginosa* colonization, while higher rankings resulted in *P. aeruginosa* displacing other members. The influence of initial cell density on three-member community coexistence and P. aeruginosa's competitive mechanisms in co-cultures is detailed in Text S1 (Fig. S8-S16).

## Discussion

Cheating, which refers to the behavior where individuals exploit the cooperative actions carried out by others [54], has been extensively studied in microbial laboratory systems in the context of toxin interference, biofilm formation [55, 56], group defense strategies [56, 57], swarming motility [58, 59], and siderophore production [60–63]. The existence of cheaters imposes a cheating load, which can potentially lead to the erosion of diversity as more advantageous forms outcompete weaker ones [64]. This leads us to consider the specific mechanisms that maintain coexistence and diversity in environments characterized by social cheating.

Previous research into coexistence within social cheating contexts predominantly delved into the interactions between distinct species. These investigations highlighted the pivotal role of spatial structures in promoting the coexistence of cooperator and cheater strains [17, 22, 65–67]. The spatial structure provided a stabilizing fitness effect by physically separating two species, thereby indirectly enhancing intraspecific competition [68]—a phenomenon consistent with ecological theory [69, 70]. Recent research has proposed alternative mechanisms that can promote coexistence and maintain biodiversity in the absence of significant spatial structure. For instance, Kelsic et al. extended the classic models of toxin production, introducing the idea of cooperative toxin degradation outside the cell [71]. Their theoretical models proposed that the opposing actions between antibiotic production and degradation counter the invasion of cheating species that cease these activities. This balance supported long-term coexistence among species with varied production and degradation abilities, even in the absence of spatial structure. This mechanism was especially pertinent in environments that generate antibiotics endogenously rather than from external introductions.

It’s crucial to recognize that social cheating is not restricted to interspecies dynamics but is also prevalent within species [72, 73]. Some theories posited that intraspecific cheating can be viewed as a constructive force, where strains collaborate by spreading antibiotic resistance, sharing public goods, and providing cross-protection against antimicrobial agents for mutual benefit and coexistence [8, 21]. In contrast to this cooperative perspective, our study revealed that, under the influence of antibiotics, intraspecific social cheating led to destructive consequences, resulting in a social dilemma characterized by intense negative interactions and the exclusion of kin members. Yet, our data indicated that the emergence of interspecific competition might alleviate these negative intraspecific inhibitions. Specifically, our findings emphasized that interspecific competition can counterbalance intraspecific exploitation and facilitate coexistence, even in scenarios where kin strains compete for limited resources within a well-mixed habitat. Based on the theoretical framework proposed by Chesson [70], we hypothesized that the presence of the regulator would exert both equalizing (convergence of fitness among competing strains with increasing species richness) and stabilizing (enhanced intraspecific competition) effects on fitness. In situations where the out-species exhibited greater competitiveness, they mitigated fitness disparities among strains through the presence of both strong damage cheaters and intermediate damage cooperators, exerting a similar “policing” effect. Such policing behaviors were widespread in the animal kingdom. For instance, insects engaged in policing to regulate worker reproduction and enhance colony reproductive efficiency [74, 75]. Among primates, macaques had been observed displaying policing behaviors to reduce conflict and strengthen social networks [76]. Our study revealed an intriguing phenomenon: even simple organisms like bacteria can also exhibit similar policing behavior and promote the coexistence of kin bacteria.

We also would like to emphasize the important role of perturbations in driving community shifts toward coexistence. While traditionally believed that disturbances lead to increased biodiversity through weakened competition [77, 78], recent theoretical and experimental evidence has challenged this notion as incomplete, highlighting the profound impact of disturbance intensity [79]. A study found that within toxic metal working fluids, individual species could not survive, but together, they thrived and efficiently degraded the fluid. This was attributed to each species’ detoxification efforts bolstering the survival and proliferation of others [80]. However, as environments were permissive—marked by reduced toxicity or the introduction of additional species—the dominant interactions tipped from cooperation toward competition, leading to unpredictable community dynamics and unstable community structure. Such community-level evolutions underlined the intrinsic connection between the impact of disturbances on communities and their intensity. Our observations aligned with this perspective, indicating that a community can only achieve coexistence when perturbations do not severely damage any of the involved members. When the antibiotic perturbation duration was extended to 48 hours, cheaters could not dominate by exploiting cooperators (Fig. S17A and S18A-B), and the introduction of an additional member did not shift this outcome (Figs S17B and S18C–E). Conversely, in environments devoid of antibiotic disturbances, coexistence remained elusive (Fig. S19). These observations highlighted a critical balance: both extended perturbations and their total absence were harmful to community coexistence. Through our study with simple bacterial communities, we provided evidence of the unexpected positive influence of subinhibitory antibiotic disturbances in shaping the coexistence of cheater and cooperator [81]. To sum up, the coexistence of cooperators and cheater strains is determined by several key conditions: (i) the emergence of perturbations during community development; (ii) competitive interactions, rather than facilitative interactions, play a crucial role in enabling community coexistence; (iii) the existence of a regulator with a growth rate similar to that of the cheater leads to reduced productivity (population size) of both the cheater and cooperator; and (iv) there are no limitations on the type of response exhibited by the regulator to antibiotics.

Based on the experimental community dynamics, we hypothesized that *P. aeruginosa*, with intermediate growth characteristics, may effectively reduce the ecological niche overlap between the two *C. testosteroni* strains by controlling their population sizes, thereby alleviating negative intraspecific interactions, and suppressing cheating behavior (Fig. 5G). Therefore, to maintain biodiversity between cheaters and cooperators, it’s important to establish an equilibrium where neither group dominated completely, which can be achieved through an external species. This external species can help maintain an intermediate level of success for both cheaters and cooperators, preventing one from monopolizing resources or ecological niches. Considering the widespread occurrence of cross-protection under toxin interference within the natural microbial world [82–84], we proposed that interspecific competition plays a vital role in maintaining biodiversity in low spatial structure habitats, such as marine ecosystems. Of course, natural microbial ecosystems are extremely complex, containing orders of magnitude more species than modeled here, as well as additional interactions such as resource competition, metabolic cross-feeding, phage invasion, and predator–prey relationships. Our serial dilution protocol mimicked a chemostat where external nutrient were consistently replenished and unused nutrients or metabolic waste were systematically removed with each growth cycle. The model primarily investigated how antibiotic sensitivity, combined with species interactions, affected the community when the growth rates of the species were uninfluenced by external environments. Consequently, our model did not encompass external resource consumption and metabolite exchanges and assumed unvarying logistic growth along with linear per capita effects of species on one another. Additionally, beyond our current scope, there are interactions, both nonmonotonic and nonadditive, that significantly influence the establishment and sustenance of microbial communities. Despite these constraints, our model effectively predicted the effects of antibiotics on the microbial community, providing valuable insights. Future research should focus on specific microbial interactions, particularly those driven by chemicals such as consumable metabolites, signaling molecules, or a combination of factors.

## Statistical analysis

Significance (^*^P < .05) was determined using the nonparametric Wilcox test. All statistical analyses were performed in R 3.1.2 (R Development Core Team, 2015). Figures were prepared using the basic R package and ggplot2.

## Conflicts of interest

The authors declare no competing interests. 

## Funding

This work was supported by the National Natural Science Foundation of China (52250056 and 52293442) and the National Key R&D Program of China (2021YFC3200603).

## Data availability

Simulation codes were available on a GitHub repository at: https://github.com/huilin970219/Coexistence_of_Kin_Bacteria. Other datasets and code generated during and/or analyzed during the current study were available from the corresponding author on reasonable request.

## Supplementary Material

SI_wrad038
