# Supplementary material for: Interspecific competition prevents the proliferation of social cheaters in an unstructured environment
Source: ISME J. 2024 Jan 10;18(1):wrad038. doi: 10.1093/ismejo/wrad038 (PMC10939377; doi:10.1093/ismejo/wrad038)
Supplement: SI_wrad038 [file si_wrad038.docx]

**Supplementary information**

**Interspecific competition prevents the proliferation of social cheaters in an unstructured environment**

Hui Lin^a,b^, Donglin Wang^a^, Qiaojuan Wang^a,b^, Jie Mao^a^, Yaohui Bai ^a,*^, Jiuhui Qu^a^

^a^ Research Center for Eco-Environmental Sciences, Chinese Academy of Sciences, Beijing 100085, China.

^b^ University of Chinese Academy of Science, Beijing 100049, China.

^*^ Corresponding author. E-mails: [yhbai@rcees.ac.cn](mailto:yhbai@rcees.ac.cn) (Y. Bai)

**Text 1 *Influence of initial cell density and pyocin production on three-member community coexistence***

Considering that initial cell density impacted coexistence in our three-member experimental community, we enhanced our theoretical model to explore this effect. We incorporated an initial cell density-dependent per capita growth rate, specifically adjusting the equation governing the dynamics of member C as

$$\frac{dC}{dt}=\gamma(D)C\left[ \frac{C}{a_{C}+C}(1-C)-\alpha_{CA}A{-\alpha}_{CB}B \right]-\mu_{C}C$$

Here, $\gamma(D)$ was modeled as a linear density-dependent function. An increase in initial cell density led to a marked rise in the growth rate of regulator C, altering the growth rate hierarchy among the three members. The resulting phase diagram identified a state of coexistence at moderate inoculum densities (Fig. S8a-c), consistent with the three-member experiment's results (Fig. 5d and S7). Detailed analysis of paired co-culture simulations further demonstrated how varying initial cell densities impact the dynamics of native cooperator and cheater. Specifically, at medium inoculum densities, regulator C, with a growth rate ranking between cooperator A and cheater B, exhibited moderate inhibition of both, avoiding complete suppression (Fig. S9a-b). These theoretical insights were corroborated by experimental observations in co-cultures of *P. aeruginosa* with KF-1 or CNB-2/∆*LuxR*. Within these co-cultures, *P. aeruginosa* inhibited the cheater CNB-2/∆*LuxR*, especially in the presence of SMX, resulting in a significant decrease in cell density by an order of magnitude (Fig. 5e, S10, and Table S3). Conversely, for the cooperator KF-1, a hormetic response activated by SMX neutralized the suppressive tendencies of *P. aeruginosa*. Hence, even under the dual pressures of competition and antibiotic exposure, its cell density, while lower compared to monoculture setups (Fig. 5f, S11, and Table S3), remained consistent with pre-antibiotic measurements (Table S4). These results illustrated that *P. aeruginosa* held a moderate competitive advantage at medium cell densities, with the cheater CNB-2/∆*LuxR* experiencing more pronounced inhibition than the cooperator KF-1.

We then delved into the mechanisms behind this competitive edge in co-cultures. *P. aeruginosa* was renowned for producing pyocins, which acted as chemical weaponry, aiding in the selective inhibition or elimination of rival strains. A quantitative assessment of pyocyanin levels, a specific pyocin from *P. aeruginosa*, revealed its increased per-cell production when co-cultured with the other two *C. testosteroni* strains for 24 h at varied initial cell densities, compared to their monocultures (Fig. S12a-b). The cultures exhibited a vivid green color indicative of elevated pyocyanin concentrations, which was more pronounced in the presence of SMX (Fig. S12c). To further determine the role of pyocyanin in providing *P. aeruginosa* with a competitive advantage, we introduced external pyocyanin into a co-culture of KF-1 and CNB-2/∆*LuxR*. While this introduction significantly decreased the growth rates of both *C. testosteroni* strains (Fig. S13), it neither significantly inhibited cheating behavior nor promoted coexistence, irrespective of the initial cell density (Fig. S14a-d). We then supplemented the three-member community with cefoperazone (256 µg/mL), a known inhibitor of pyocyanin production. Observations revealed a 90% reduction in pyocyanin production with CFP present across all three initial cell densities (Fig. S15). However, upon introducing CFP, the previously observed coexistence scenario disappeared (Fig. S16), further proving the crucial role of pyocyanin in bolstering *P. aeruginosa*’s competitive edge. To sum up, the presence of *P. aeruginosa* and its inherent ability to produce the biochemical weapon, pyocyanin, proved vital for maintaining coexistence.

**Text 2 *Construction of mutants carrying LuxR and Sul1***

All primer pairs used for gene complementation are listed in Table S6. To construct the mutant carrying the *LuxR* gene, we amplified the *C. testosteroni LuxR* gene using PCR with the *LuxR*-comF and *LuxR*-comR primers. To construct the mutant complemented with the SMX resistance gene *Sul1*, we amplified the *Sul1* gene using PCR with the Sul1a-F and Sul1b-R primers. The In-Fusion technique was used to clone the DNA fragment into the *Xba*I and *EcoR*I sites of the tetracycline-resistant plasmid vector pRK415 to yield pRK415-*LuxR* and pRK415-*Sul1*. The plasmids were then respectively transformed into the *Escherichia* *coli* WM3064 via electroporation and conjugation with the *P. aeruginosa* PAO1. The transconjugants were selected on Luria-Bertani (LB) plates containing tetracycline (10 μg/mL). The PAO1-pRK415*LuxR* and PAO1-pRK415*Sul1* mutants were confirmed by PCR using the 415-F/R primers.

**Text 3 *Methodology for sulfamethoxazole (SMX) measurement by ultra-performance liquid chromatography-tandem mass spectrometry (UPLC-MS/MS)***

SMX analysis was performed by LC-MS. A binary gradient system was applied, consisting of mobile phase A (0.1% formic acid in Milli-Q water) and mobile phase B (methanol (MS analysis grade)) as separate analytes in a Hypersil GOLD column (2.1 mm × 100 mm, particle size 1.8 μm, Thermo Scientiﬁc, Bellefonte, USA). The solvent gradient was 5% B to 95% B in 7 min, with maintenance at 95% B to 15 min. The high-performance liquid chromatograph (HPLC) was connected to an electrospray ionization probe (ESI) of a TSQ Altis Quantum Ultra triple quadrupole mass spectrometer (Thermo Scientiﬁc, San Jose, CA, USA) operated under unit resolution in the selected reaction monitoring (SRM) mode. Nitrogen was used as the sheath gas (60 arbitrary units) and auxiliary gas (15 arbitrary units), and argon was used as the collision gas (1.5 m Torr). Analyses were performed at a spray voltage of +3 800 V (positive mode) and capillary temperature of 35 ℃. Quality control was performed for each experiment using batch samples selected randomly by spike recovery experiments (spiking at 50 μg/L), which showed a maximum relative standard deviation (RSD) range within 2.36%.

**Text 4** ***Pyocyanin accumulation in cultures***

The *P. aeruginosa* PAO1 monoculture, mixed *C. testosteroni* KF-1 or CNB-2/Δ*LuxR* cultures, and three-member cultures in MSM were collected at the exponential phase (48 h). Excreted pyocyanin by *P. aeruginosa* was quantified based on the presence of a pink to deep red color in an acidic solution. In brief, 2.5 mL of supernatant from the NB broth was mixed with 1.5 mL of chloroform after 24 h of growth. Pyocyanin in the chloroform phase was then extracted into 0.5 mL of 0.2 mol/L hydrochloric acid (HCI). After centrifugation at 5,000 ×g for 2 min, absorbance of the top layer was measured at 520 nm. Concentrations of pyocyanin were expressed as micrograms of pyocyanin per milliliter of supernatant, determined by multiplying the absorbance at 520 nm (A520 nm) by 17.1. The cell density in supernatant normalized per-cell estimates.

**
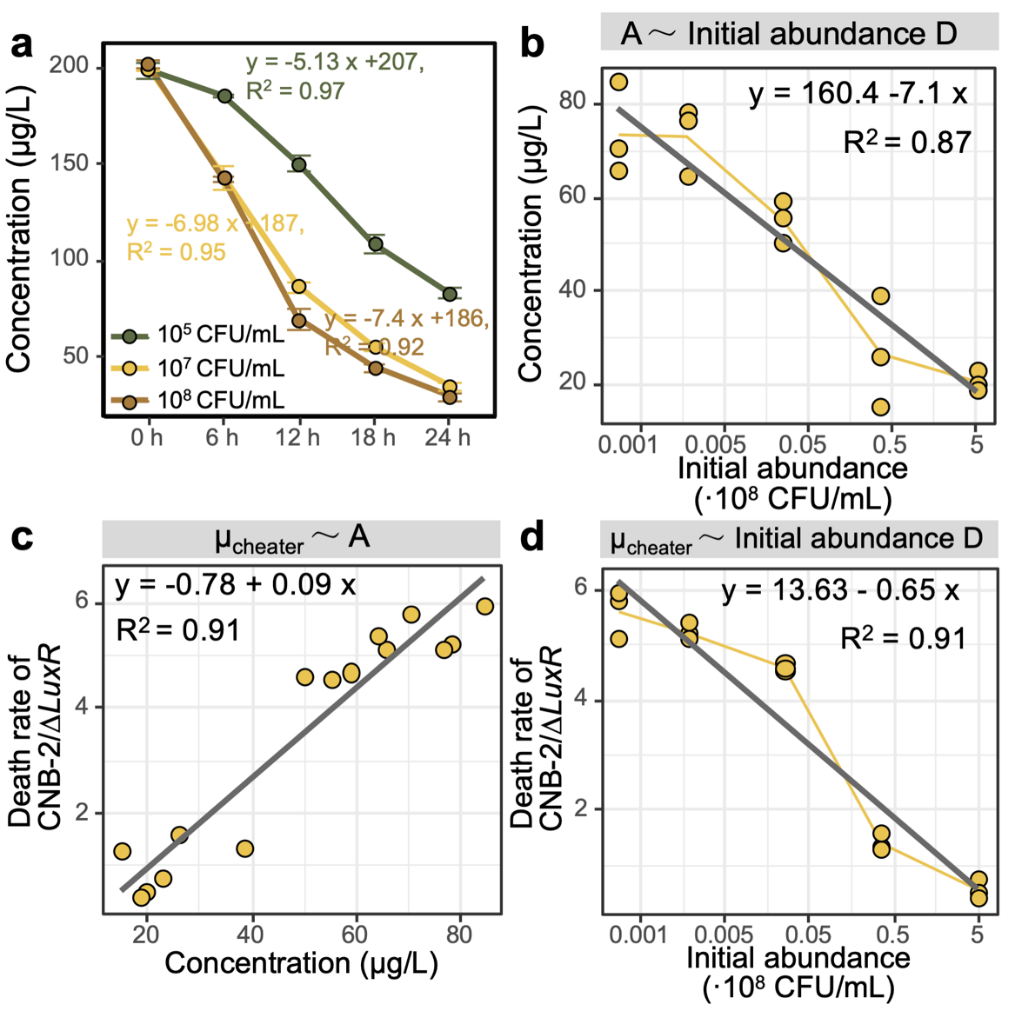
**

**Figure S1** *Comamonas testosteroni* KF-1 strain can detoxify sulfamethoxazole (SMX) and allow subsequent growth of sensitive CNB-2/Δ*LuxR*. (**a**) Changes in SMX concentration were evaluated in co-cultures of KF-1 and CNB-2/Δ*LuxR* with initial inoculum densities ranging from 10^5^ to 10^8^ CFU/mL. Subfigures (**b**) and (**c**) showed the dynamics of antibiotic clearance and its effect on cheater cell mortality. (**b**) The cooperator’s capability to clear the antibiotic intensified with higher initial cell densities. As a result, (**c**) the rapid reduction in antibiotic concentration led to decreased average cheater mortality within the interference cycle. (**d**) 24-hour antibiotic-induced cheater mortality linearly decreased with higher initial cell densities. Death rate was determined by dividing the decrease in cell density by the time elapsed following 24-hour antibiotic exposure. Each panel was supplemented with a regression line and corresponding R^2^ value. In all regression analyses, the T-test yielded *p*-values less than 0.05.


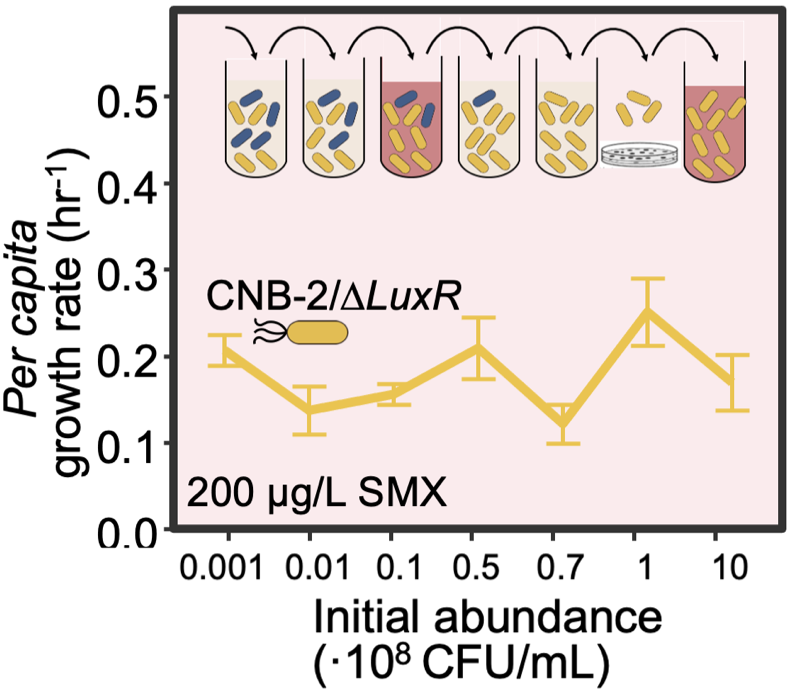


**Figure S2** Investigating the potential for CNB-2/Δ*LuxR* to acquire resistance through horizontal gene transfer from KF-1. Post-sulfamethoxazole (SMX) exposure CNB-2/Δ*LuxR* cells, co-cultured with KF-1, were isolated and their growth rates evaluated in a new medium containing 200 µg/L SMX (n = 6).


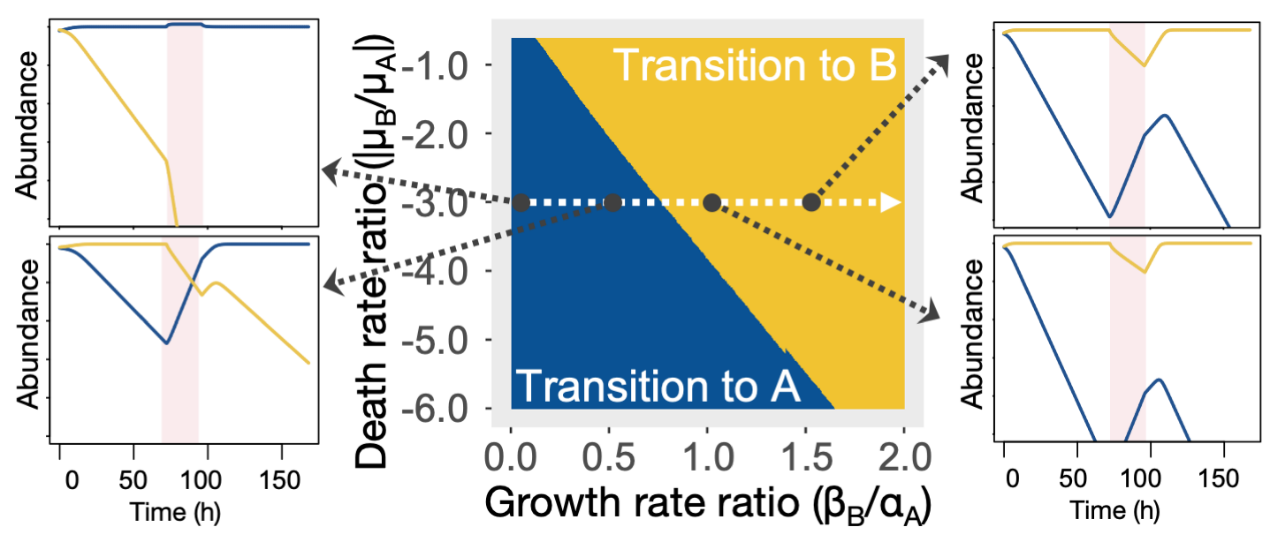


**Figure S3** The phase diagram and the representative time series depicted model predictions regarding the impact of antibiotic exposure on a kin bacterial community. As the death rate ratio (|μ_B_/μ_A_|) diminished, intrinsic growth rate differences led to the predominance of cheater B (progressing from left to right).


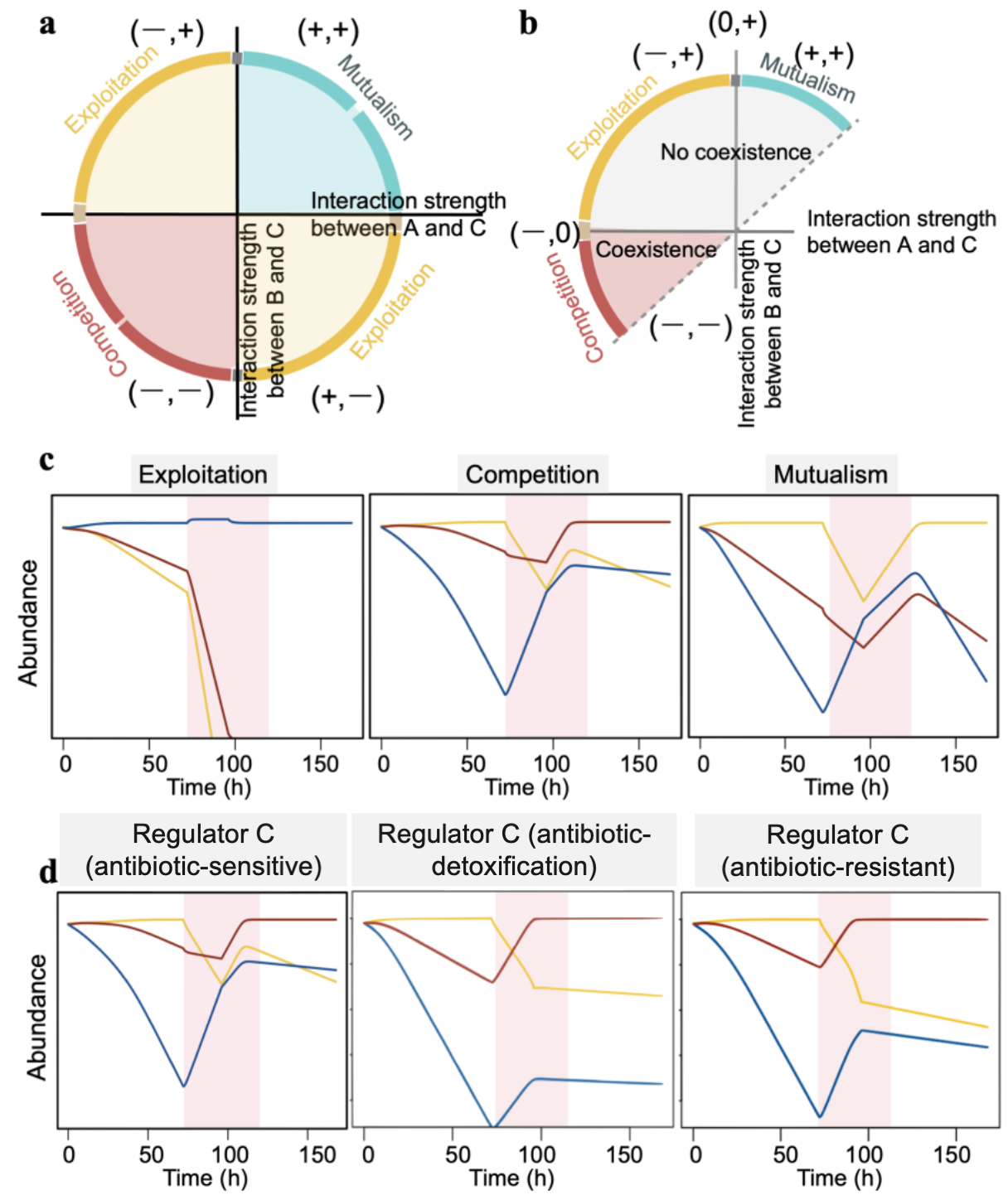


**Figure S4** Time series illustrating community dynamics. (**a**) Bidirectional interactions between third and native member (α_AC,_ α_BC_) were classified as competition (−/−), exploitation (−/+), or mutualism (+/+). Subfigures (**b**) and (**c**) demonstrate that community coexistence is driven by competitive interactions rather than facilitative interactions. Subfigure (**d**) shows the community dynamic with a fixed faster growth rate of regulator C [γ_C_ = β_B_ = 0.75] and pairwise negative interactions, considering strains with varying responses to three antibiotics: antibiotic-sensitive, antibiotic-resistant, and intrinsically resistant strains.


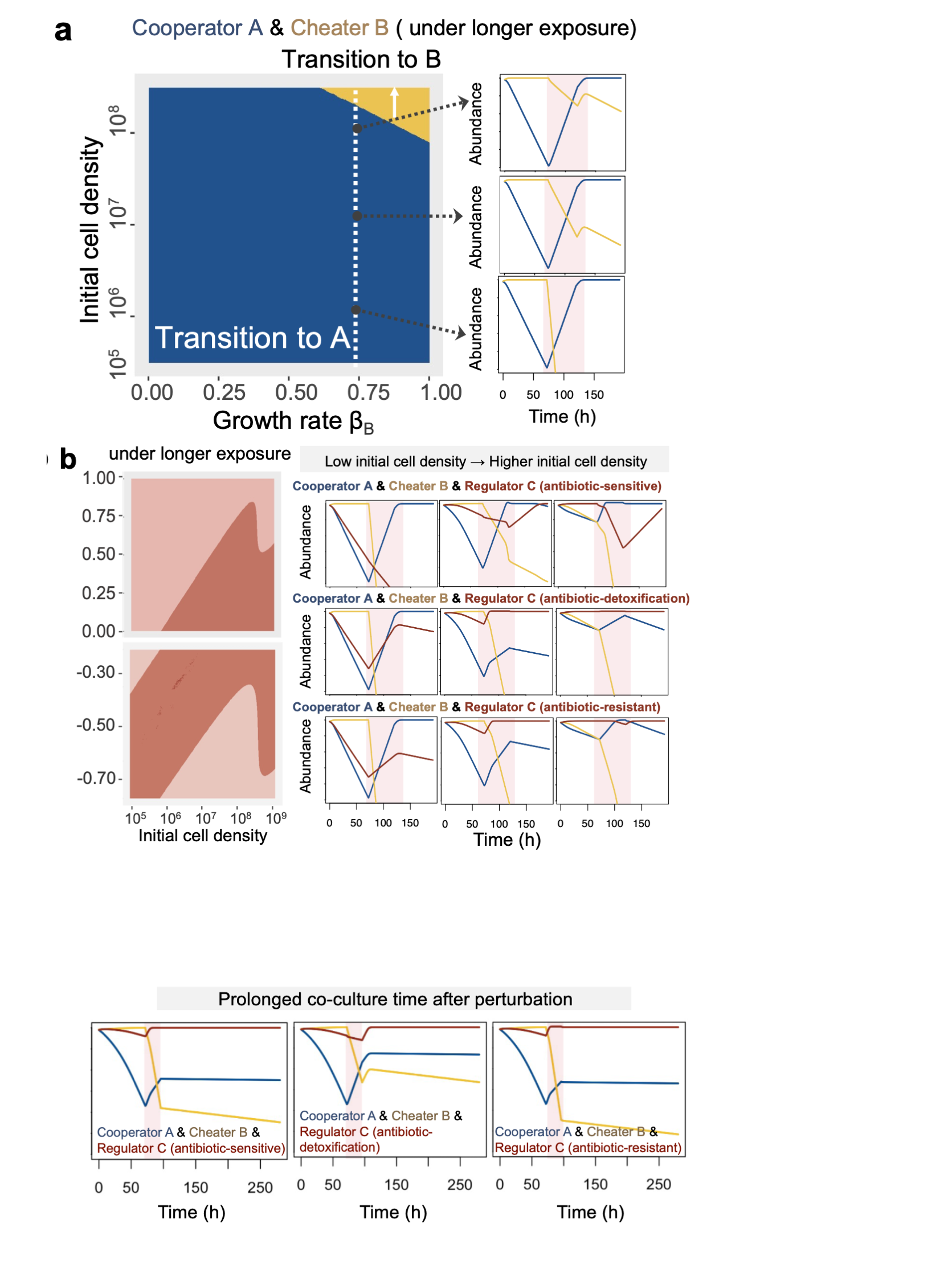


**Figure S5** The sustained equilibrium in the three-member community after antibiotic exposure in the absence of additional disturbances (T = 264 h).


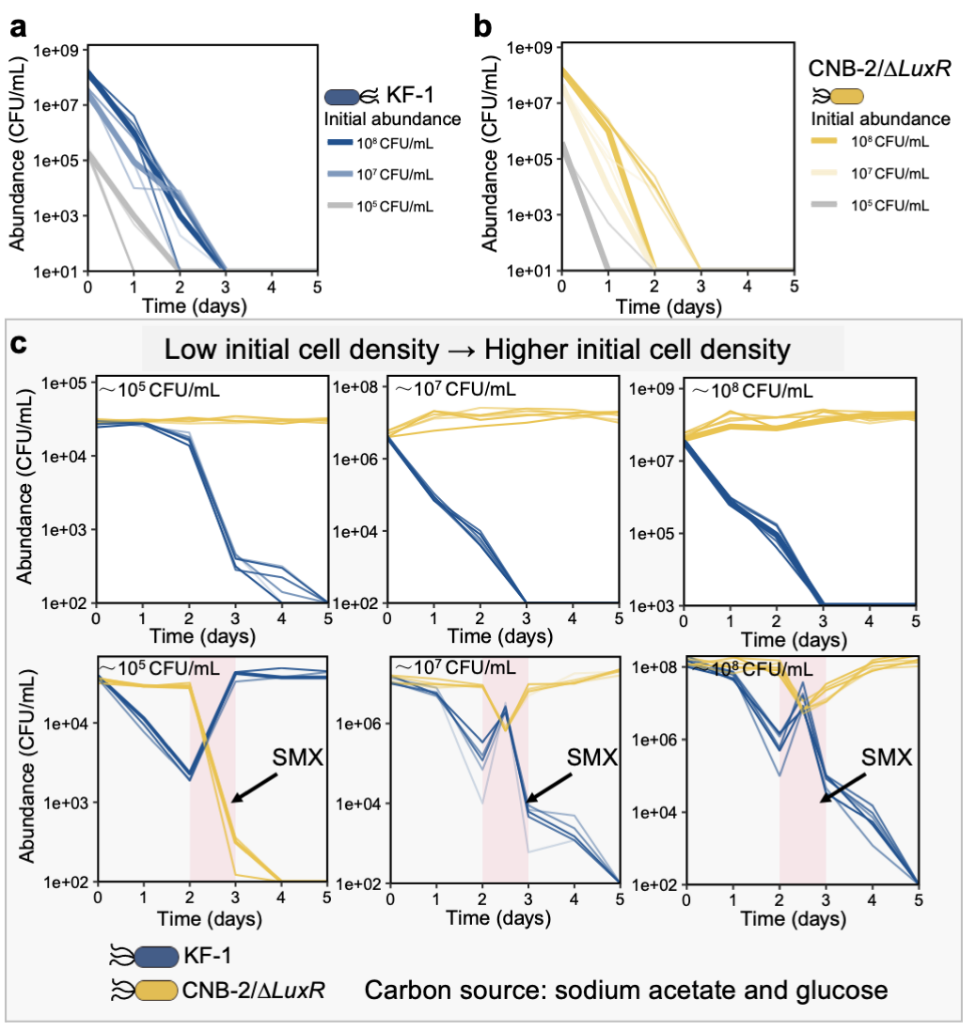


**Figure S6** (**a**) KF-1 and (**b**) CNB-2/∆*LuxR* exhibited a rapid decline in cell density when cultivated in a glucose-only medium. As a result, (**c**) the coexistence dynamics of the two *Comamonas testosteroni* strains in dual carbon sources (sodium acetate and glucose) were consistent with the dynamics observed in a sodium acetate-only environment. (n = 6).


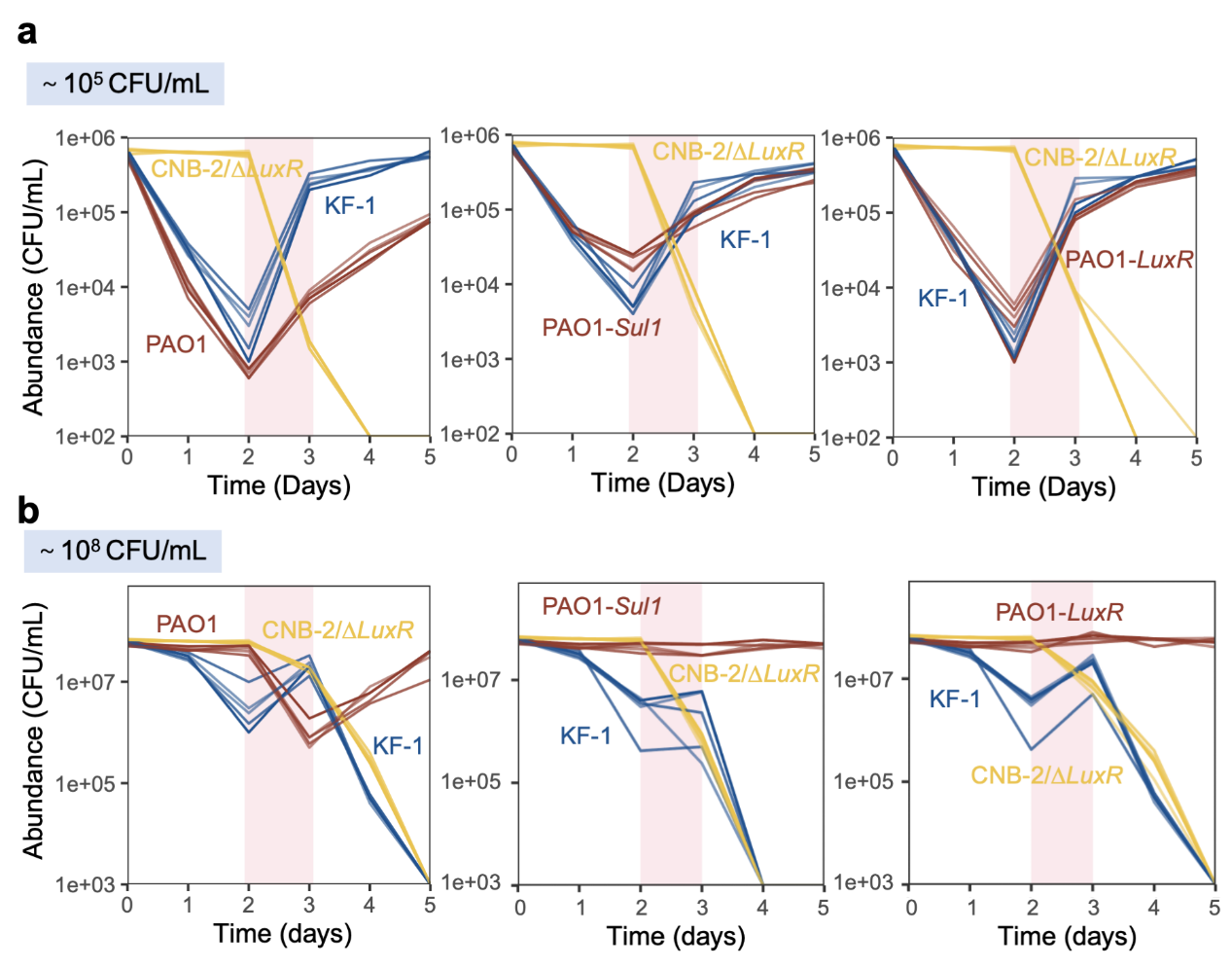


**Figure S7** Effects of lower (10^5^ CFU/mL, **a**) and higher (10^8^ CFU/mL, **b**) initial density on three-member community dynamics in the presence of 200 µg/L sulfamethoxazole: *Comamonas testosteroni* KF-1, *Comamonas testosteroni* CNB-2/Δ*LuxR*, and *Pseudomonas aeruginosa* PAO1 (or PAO1-*Sul1* or PAO-*LuxR*). Using the previous 5-day serial dilution protocol, we assessed synthetic community dynamics (n = 6).

**
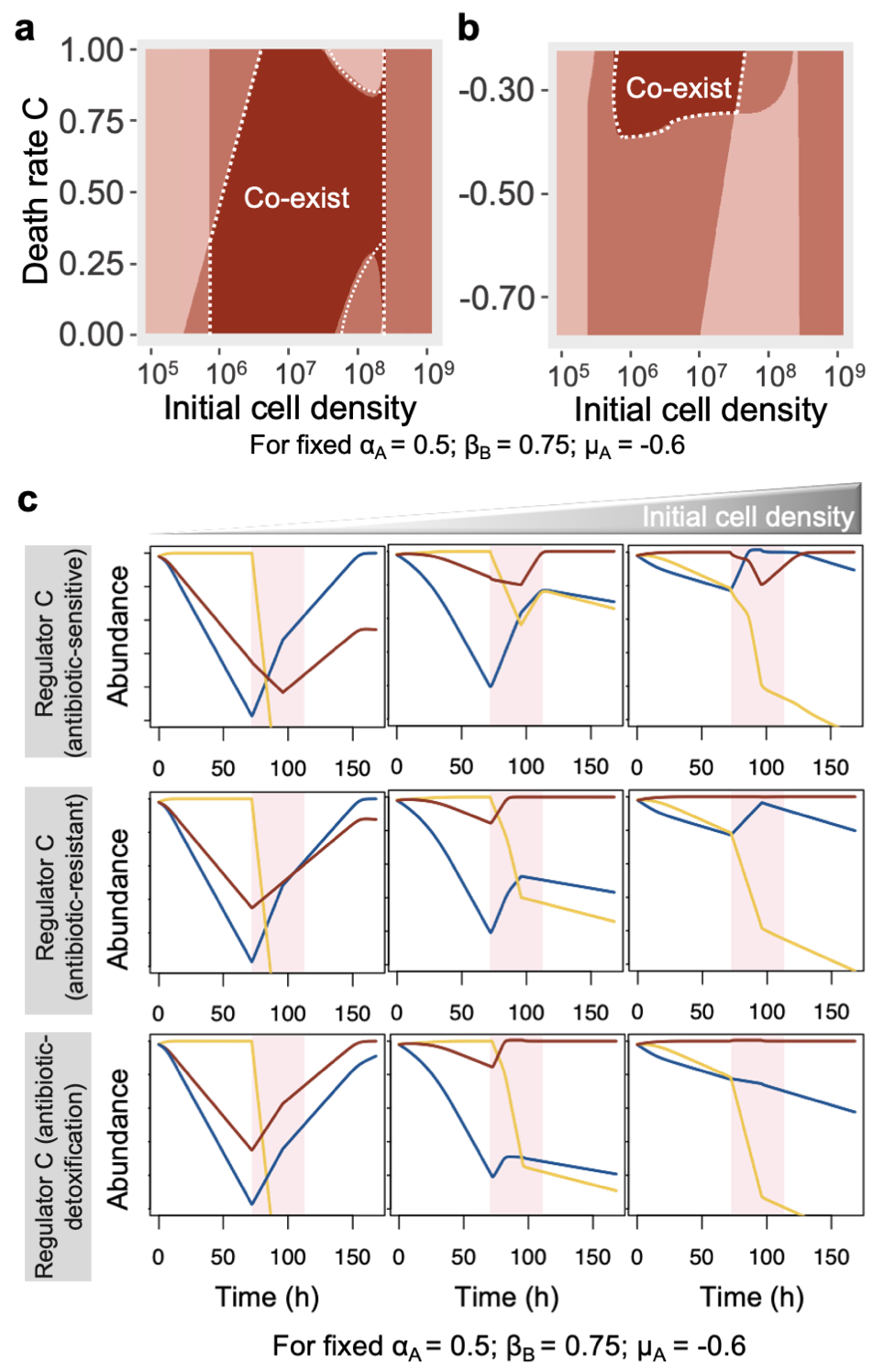
**

**Figure S8** Phase diagrams (**a**) and (**b**), along with time series (**c**), showed the impact of initial cell densities and antibiotic reactions on the community coexistence. Potential coexistence was observable at moderate inoculum densities (~ 10^7^ CFU/mL), regardless response to antibiotic exposure of member C.


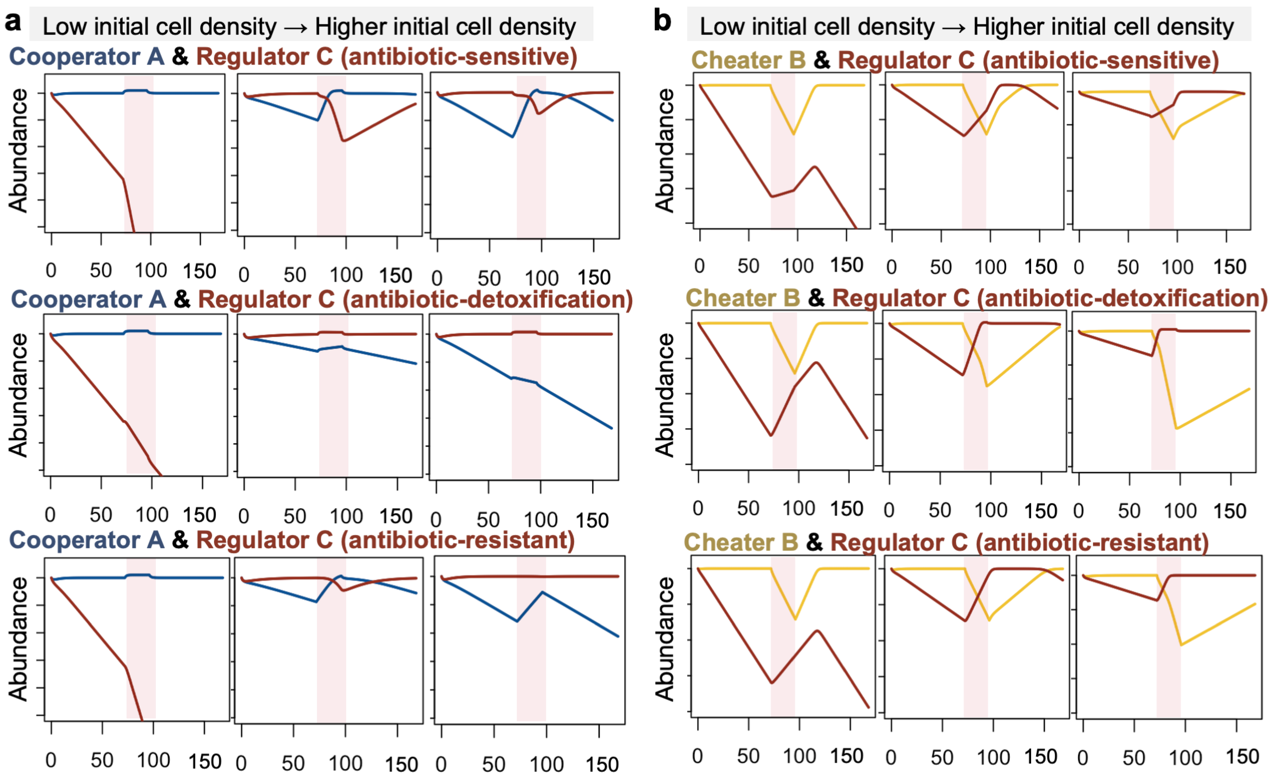


**Figure S9** Modeling growth dynamics in paired cultures: (**a**) interactions between members A and C; (**b**) interactions between members B and C, employing a model with a density-dependent function for member C’s growth rate.


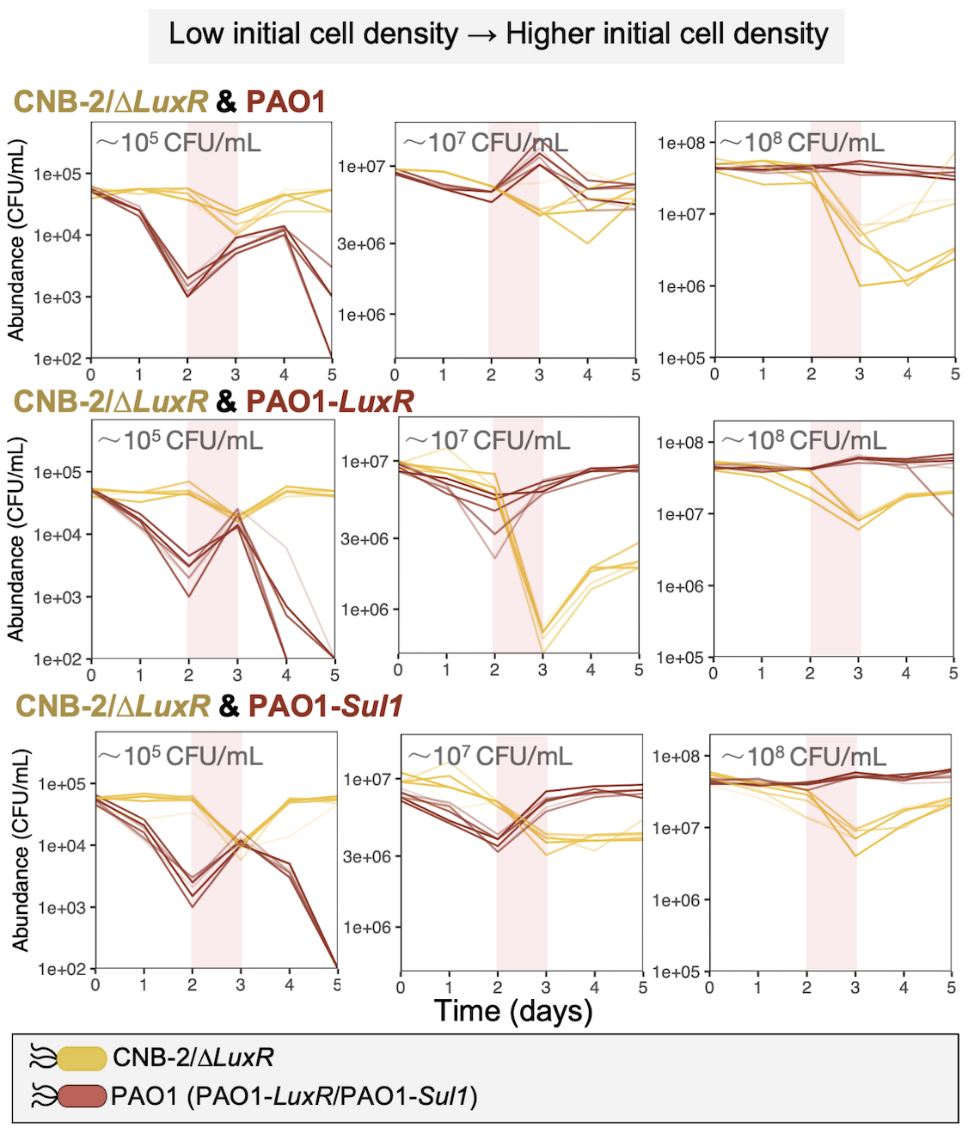


**Figure S10** Experimental dynamics of co-culture involving three phenotypic strains of PAO1 and CNB-2/∆*LuxR* at varying initial cell densities (n = 6). Daily, cultures were diluted by transferring 1/10 of the previous day’s volume to fresh medium, maintaining a consistent volume.


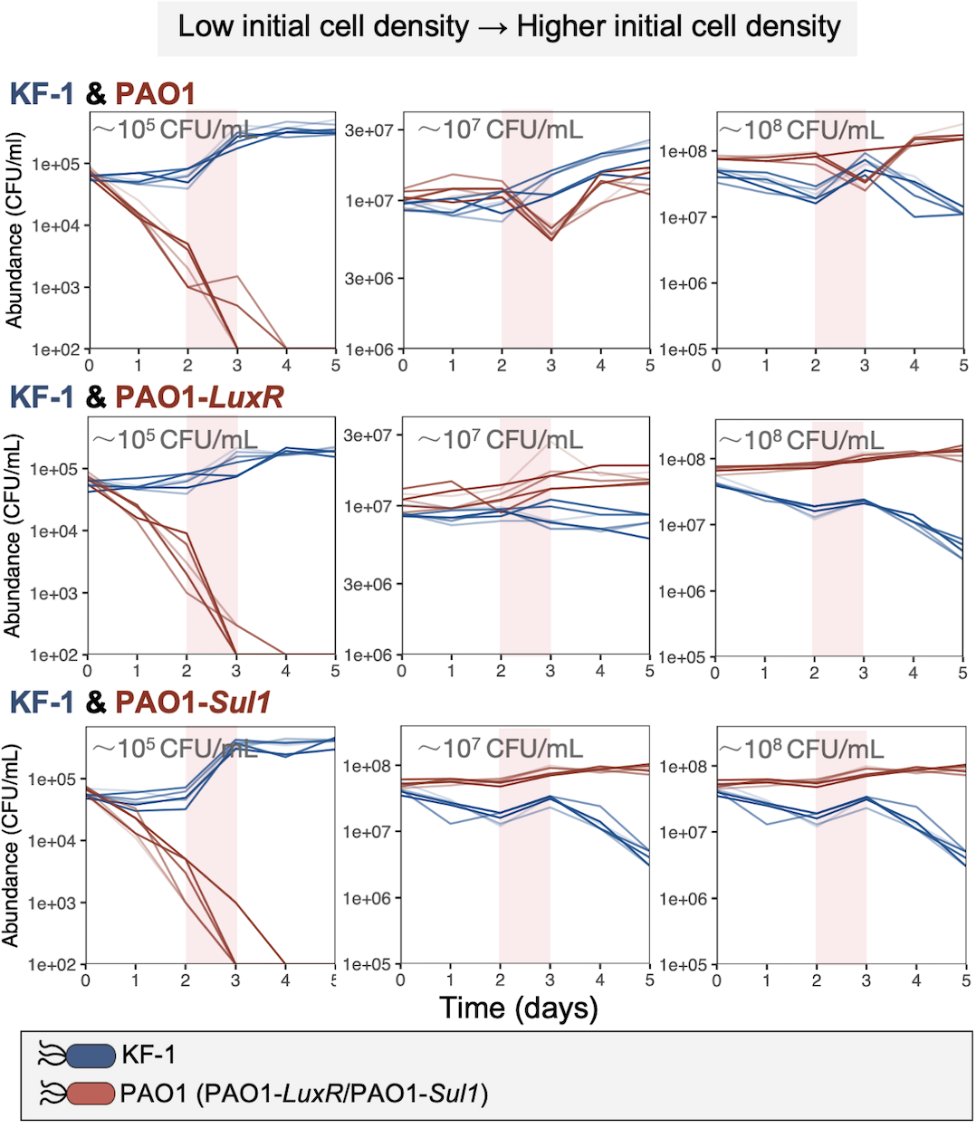


**Figure S11** Experimental dynamics of co-culture involving three phenotypic strains of PAO1 and KF-1 at varying initial cell densities (n = 6). Daily, cultures were diluted by transferring 1/10 of the previous day’s volume to fresh medium, maintaining a consistent volume.


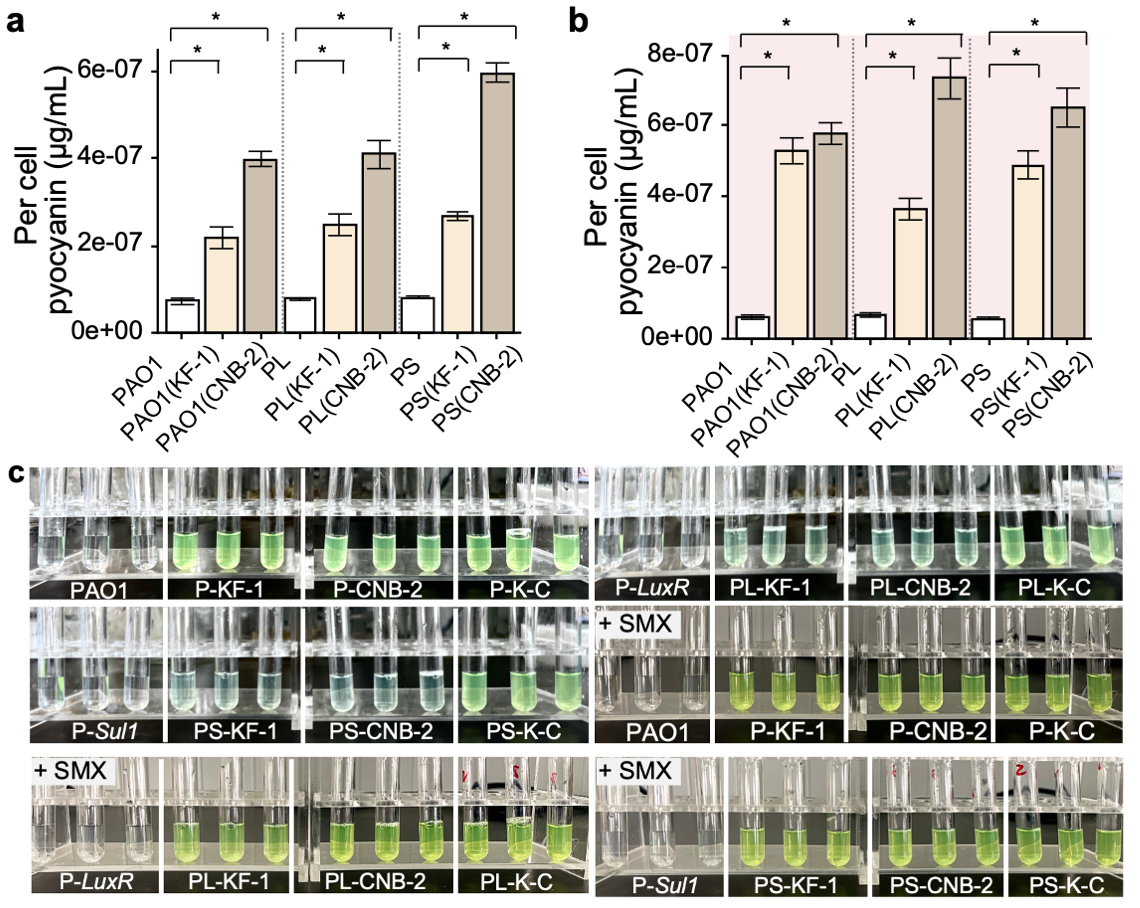


**Figure S12** Enhanced per-cell pyocyanin production in *Pseudomonas aeruginosa* when co-cultured with competitor strains. (**a**) Observable increase in production conferring a competitive advantage (n = 12; initial cell densities at ~ 10^7^ and ~ 10^8^ CFU/mL). (**b**) The effect was accentuated in environments with 200 µg/L sulfamethoxazole (n = 12; initial cell densities at ~ 10^7^ and ~ 10^8^ CFU/mL). (**c**) Color comparison of different strain cultures. **p* < 0.05.


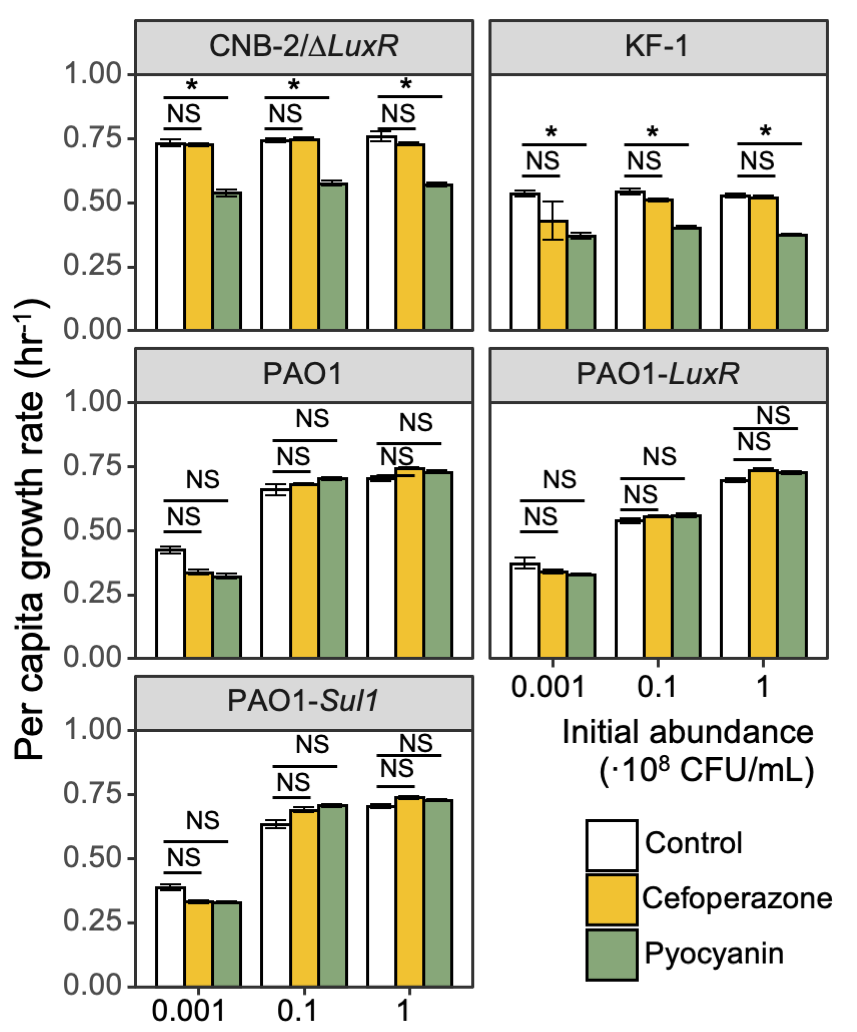


**Figure S13** Impact of pyocyanin (6 µg/mL) and cefoperazone (256 µg/mL) on the growth rates of *Comamonas testosteroni* and *Pseudomona aeruginosa* (n = 6). Significance levels (ns, *p* > 0.05; **p* < 0.05) were assessed using the Wilcox-test.


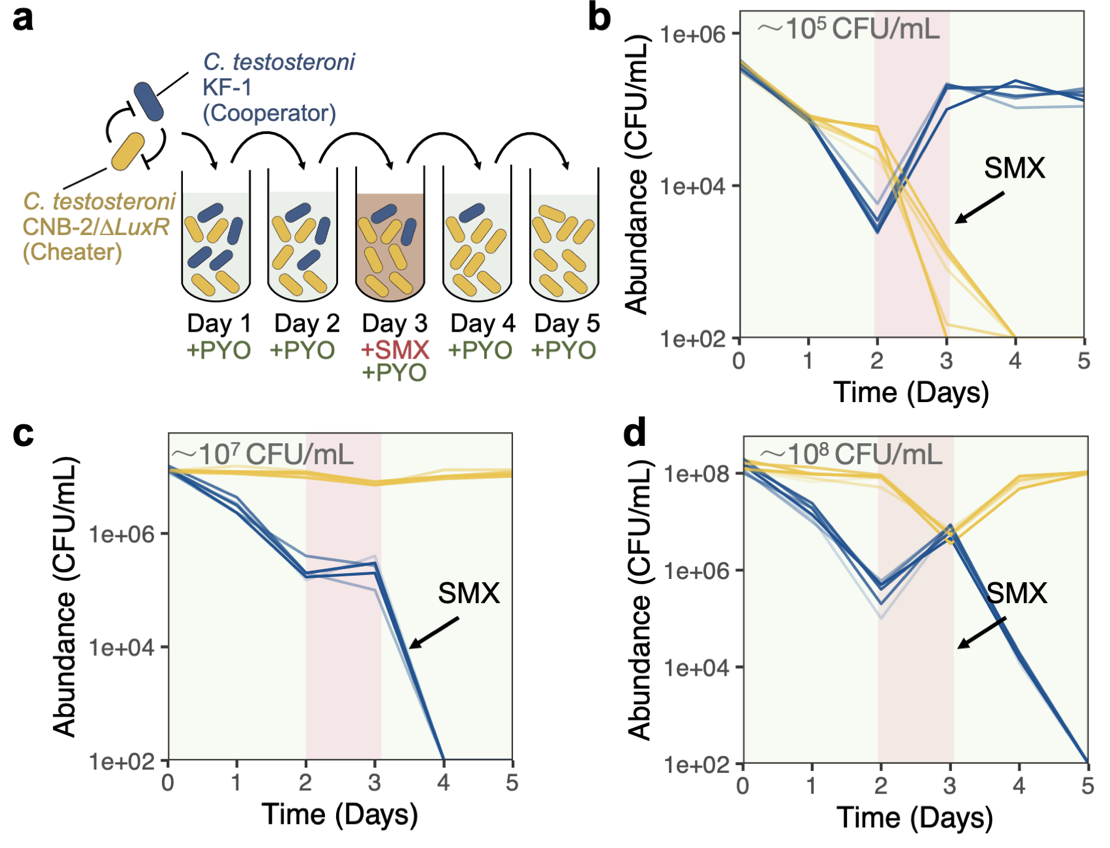


**Figure S14** Impact of external pyocyanin (PYO) on coexistence in the CNB-2/∆*LuxR* and KF-1 co-culture. (**a**) Co-culture of CNB-2/∆*LuxR* and KF-1 underwent daily dilutions at a factor of 10 (1/10 of the previous day’s culture transferred to fresh medium, with a constant volume). Subfigures (**b**)**-**(**d**) demonstrated that at the higher initial cell density, fast-growing CNB-2/∆*LuxR* dominated and drove slow-growing KF-1 to extinction (n = 6). Adding external PYO did not alter this community outcome or foster coexistence.


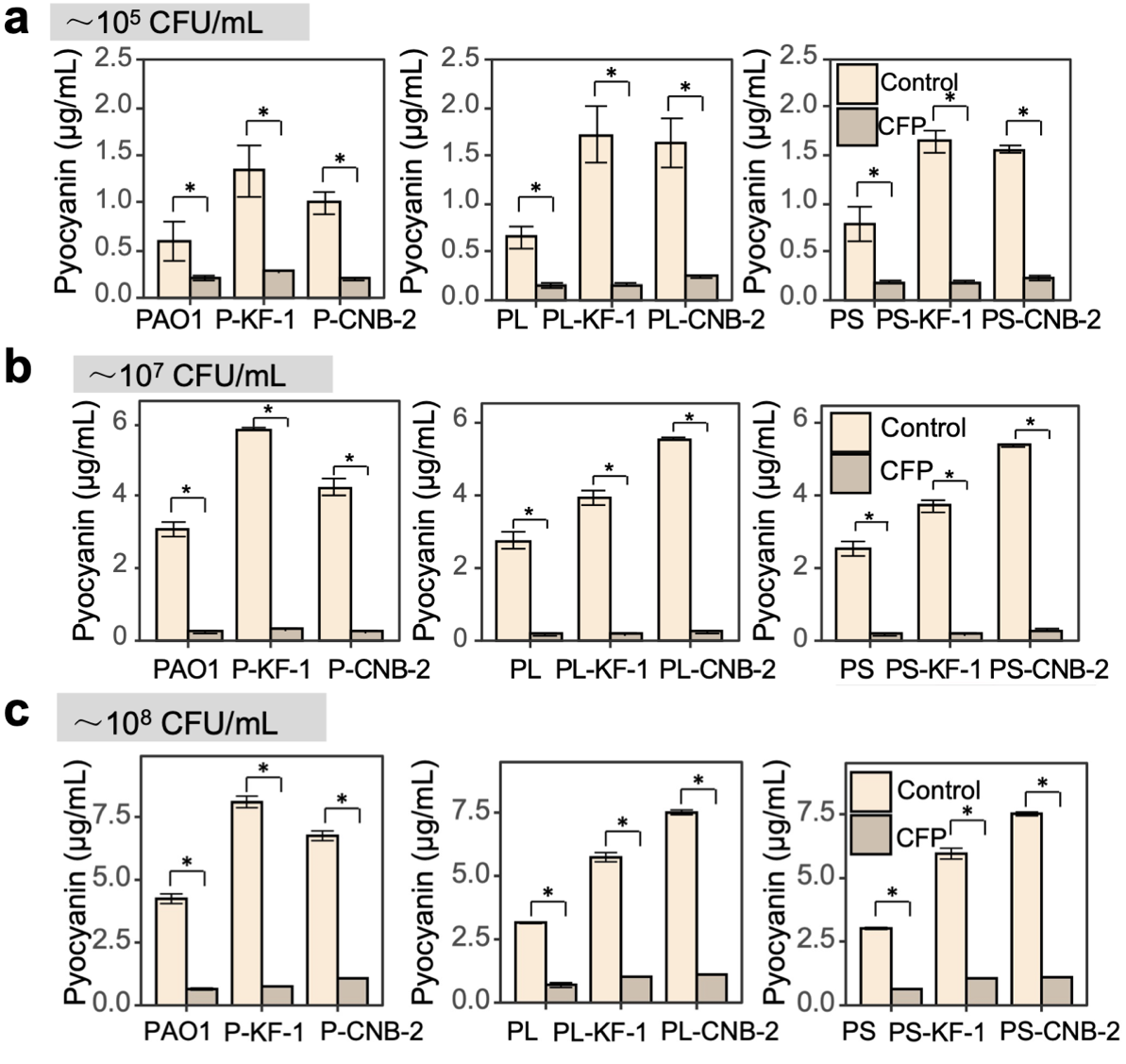


**Figure S15** Inhibition of cefoperazone (CFP, 256 µg/mL) to the production of pyocyanin at inoculum densities of 10^5^, 10^7^, and 10^8^ CFU/mL, respectively (n = 6). Significance levels (ns, *p* > 0.05; **p* < 0.05) were assessed using the Wilcox-test.


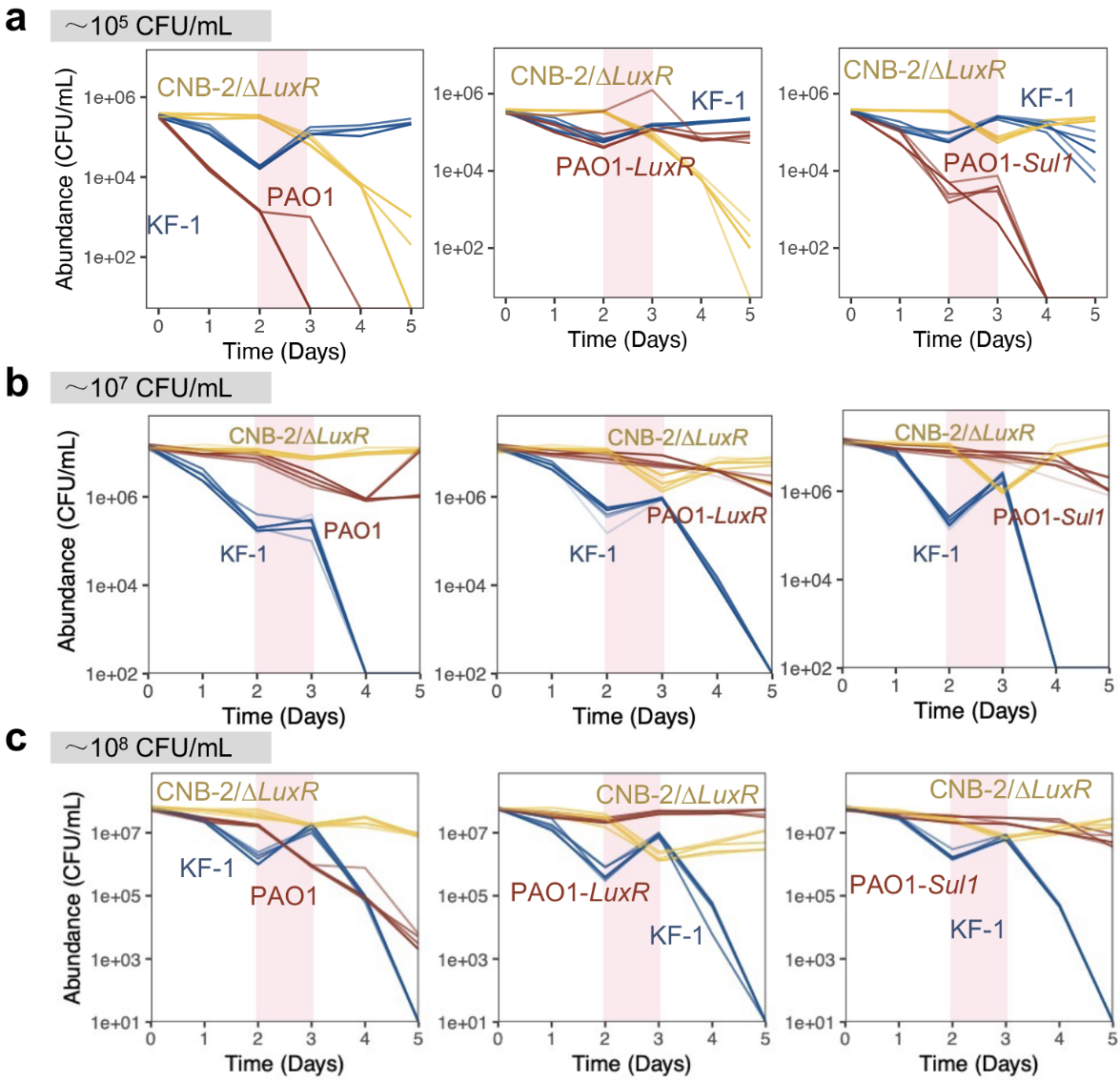


**Figure S16** Effects of cefoperazone (CFP, 256 µg/mL), an inhibitor of pyocyanin production, on the community dynamics of a three-member system at inoculum densities of approximately (**a**) 10^5^, (**b**) 10^7^, and (**c**) 10^8^ CFU/mL, respectively (n = 6).


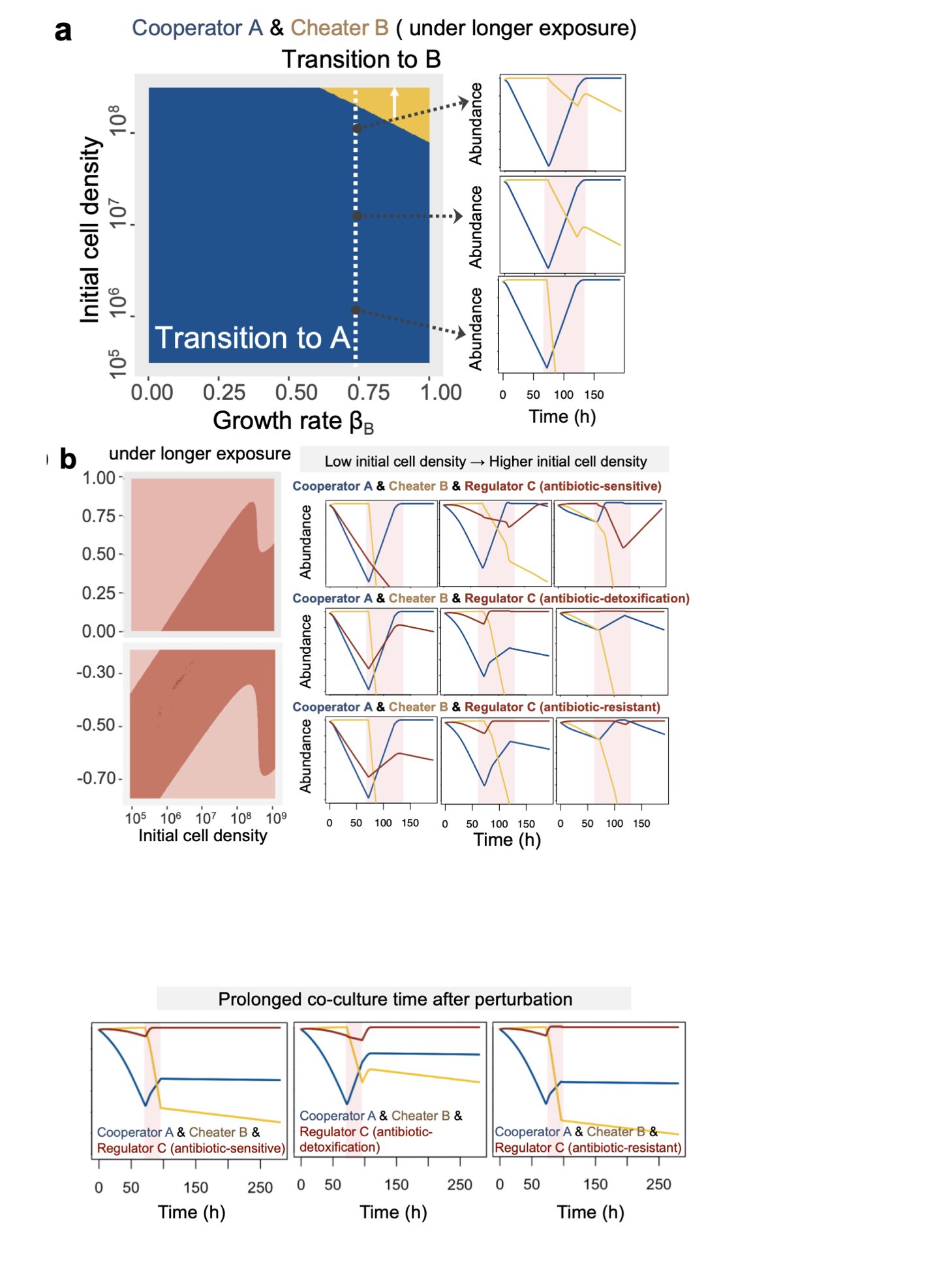


**Figure S17** Modeling dynamics of community behavior during prolonged antibiotic exposure. The phase diagram and associated simulated time series depicted the responses of (**a**) two-member and (**b**) three-member communities over a 48-hour antibiotic exposure. The period of antibiotic exposure was highlighted in pink, indicating the 48-hour cycle.


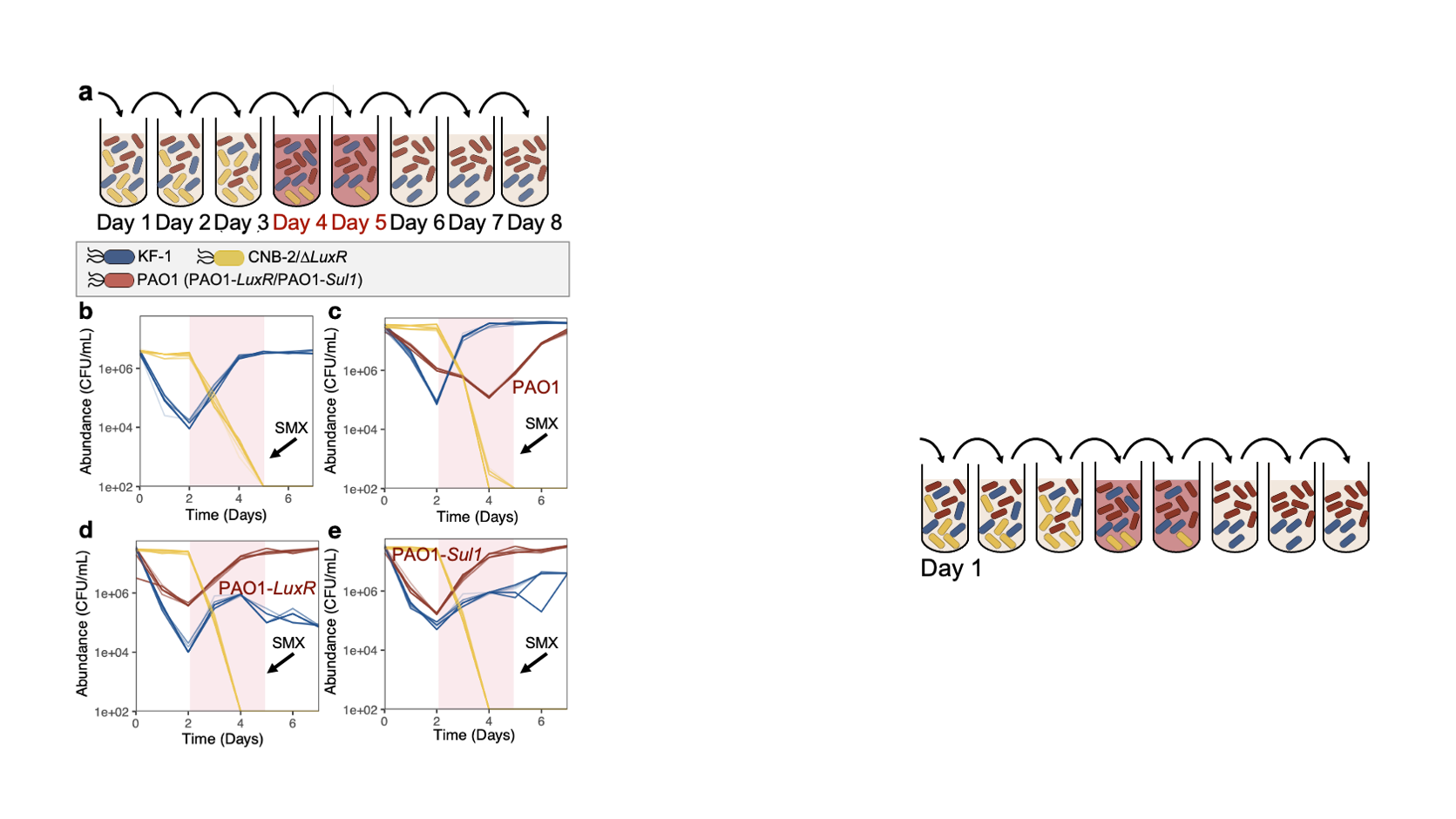


**Figure S18** Experimental dynamics of a community under prolonged antibiotic exposure carried out at a medium inoculum density (~ 10^7^ CFU/mL) using 8-day serial dilution (**a**). Daily 10-fold dilution of co-cultures in two-member communities (**b**) KF-1 and CNB-2/∆*LuxR*, and three-member communities involving KF-1, CNB-2/∆*LuxR* and (**c**) PAO1, (**d**) PAO1-*LuxR*, or (**e**) PAO1-*Sul1* (n = 6). The period of sulfamethoxazole exposure was highlighted in pink, indicating the 48-hour cycle.


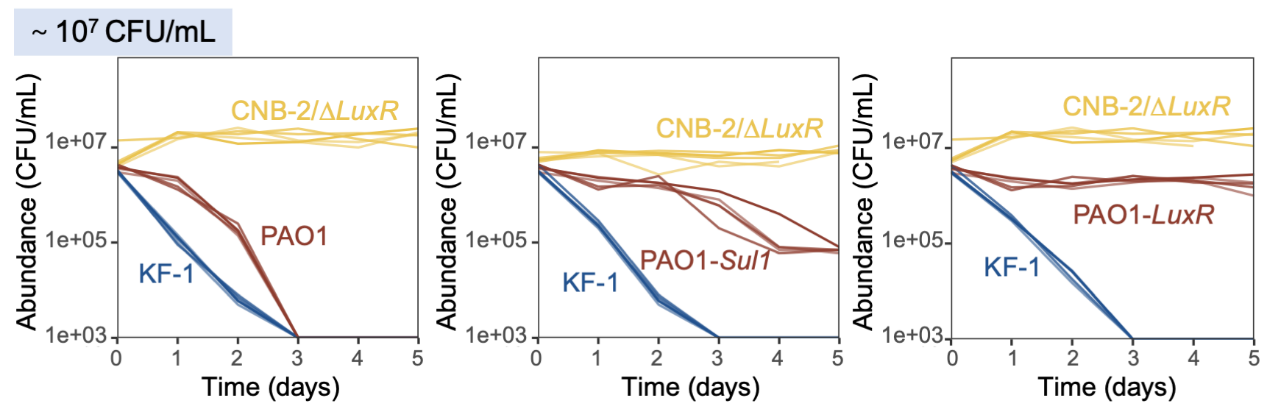


**Figure S19** Community dynamics in the absence of sulfamethoxazole disturbance: *Comamonas testosteroni* KF-1, *Comamonas testosteroni* CNB-2/Δ*LuxR*, and *Pseudomonas aeruginosa* PAO1, PAO1-*Sul1*, or PAO1-*LuxR* (n = 6).

**Table S1** The average growth rate of the community members at different initial inoculum density, referred to the population growth rate normalized by the initial population size.

| Initial Density | KF-1 | CNB-2  /∆*LuxR* | | PAO1 | | PAO1-*Sul1* | PAO1-*LuxR* |
| --- | --- | --- | --- | --- | --- | --- | --- |
| 1×10^5^ | 0.54 | 0.73 | 0.43 | | 0.37 | | 0.33 |
| 5×10^5^ | 0.55 | 0.74 | 0.48 | | 0.43 | | 0.35 |
| 7×10^5^ | 0.56 | 0.71 | 0.55 | | 0.51 | | 0.47 |
| 1×10^6^ | 0.58 | 0.78 | 0.42 | | 0.40 | | 0.41 |
| 1×10^7^ | 0.53 | 0.77 | 0.51 | | 0.51 | | 0.47 |
| 3×10^7^ | 0.50 | 0.75 | 0.65 | | 0.65 | | 0.60 |
| 5×10^7^ | 0.50 | 0.74 | 0.61 | | 0.60 | | 0.53 |
| 7×10^7^ | 0.56 | 0.75 | 0.70 | | 0.66 | | 0.58 |
| 9×10^7^ | 0.53 | 0.75 | 0.72 | | 0.73 | | 0.70 |
| 1×10^8^ | 0.53 | 0.76 | 0.72 | | 0.72 | | 0.68 |
| 5×10^8^ | 0.52 | 0.73 | 0.76 | | 0.77 | | 0.74 |
| 7×10^8^ | 0.50 | 0.74 | 0.77 | | 0.79 | | 0.76 |
| 1×10^9^ | 0.51 | 0.82 | 0.69 | | 0.69 | | 0.71 |

**Table S2** The average growth rate of the community members at different initial inoculum density in the presence of sulfamethoxazole, referred to the population growth rate normalized by the initial population size.

| Initial Density | KF-1 | CNB-2  /∆*LuxR* | | PAO1 | | PAO1-*Sul1* | PAO1-*LuxR* |
| --- | --- | --- | --- | --- | --- | --- | --- |
| 1×10^5^ | 0.59 | 0.12 | 0.17 | | 0.42 | | 0.50 |
| 5×10^5^ | 0.55 | 0.19 | 0.21 | | 0.43 | | 0.45 |
| 7×10^5^ | 0.54 | 0.18 | 0.21 | | 0.50 | | 0.59 |
| 1×10^6^ | 0.62 | 0.13 | 0.20 | | 0.46 | | 0.62 |
| 1×10^7^ | 0.63 | 0.26 | 0.21 | | 0.57 | | 0.71 |
| 3×10^7^ | 0.55 | 0.24 | 0.23 | | 0.60 | | 0.70 |
| 5×10^7^ | 0.67 | 0.26 | 0.16 | | 0.57 | | 0.75 |
| 7×10^7^ | 0.70 | 0.32 | 0.19 | | 0.67 | | 0.79 |
| 9×10^7^ | 0.70 | 0.28 | 0.27 | | 0.73 | | 0.75 |
| 1×10^8^ | 0.68 | 0.31 | 0.20 | | 0.71 | | 0.77 |
| 5×10^8^ | 0.60 | 0.26 | 0.28 | | 0.77 | | 0.77 |
| 7×10^8^ | 0.65 | 0.25 | 0.30 | | 0.79 | | 0.79 |
| 1×10^9^ | 0.74 | 0.37 | 0.21 | | 0.69 | | 0.75 |

**Table S3** Differences [*p-*value] in daily population densities of *Comamonas testosteroni* strains under monoculture and co-culture conditions, with *p*-values calculated using the Wilcox-test.

|  | **KF-1:KF-1(PAO1)** | **KF-1:KF-1(PAO1-*Sul1*)** | **KF-1:KF-1(PAO1-*LuxR*)** |
| --- | --- | --- | --- |
| Day 0 | 0.1520242 | 0.1235158 | 0.07141016 |
| Day 1 | 0.4200704 | 0.6878846 | 0.6863111 |
| Day 2 | 0.145251 | 0.1255303 | 0.1914184 |
| Day 3 | 0.004060097 | 0.004266725 | 0.004266725 |
| Day 4 | 0.004771822 | 0.004998125 | 0.004922036 |
| Day 5 | 0.004998125 | 0.004998125 | 0.004697697 |
|  | **CNB-2:CNB-2(PAO1)** | **CNB-2:CNB-2(PAO1-*Sul1*)** | **CNB-2:CNB-2(PAO1-*LuxR*)** |
| Day 0 | 0.2934668 | 0.4408765 | 0.8088656 |
| Day 1 | 0.002268132 | 0.285844 | 0.05620438 |
| Day 2 | 0.002724532 | 0.05574205 | 0.004697697 |
| Day 3 | 0.03637858 | 0.004998125 | 0.004697697 |
| Day 4 | 0.03869781 | 0.004998125 | 0.004697697 |
| Day 5 | 0.03755444 | 0.004771822 | 0.004624229 |

(Groups before and after ‘:’ used for the Wilcox-test)

**Table S4** Significance [*p-*value] of change in population densities of *Comamonas testosteroni* strains before (Day 1, Day 2 and Day 3) and after (Day 3, Day 4 and Day 5) antibiotic interference under both monoculture and co-culture conditions using dilution culture protocols. The *p*-values were calculated using the Wilcox-test.

| **Member** | **Wilcox-test *p* value** |
| --- | --- |
| CNB-2/∆*LuxR* | 2.17E-06 |
| CNB-2/∆*LuxR* (PAO1-*LuxR*) | 3.00E-07 |
| CNB-2/∆*LuxR* (PAO1) | 4.71E-06 |
| CNB-2/∆*LuxR* (PAO1-*Sul1*) | 3.03E-07 |
| KF-1 | 6.11E-07 |
| KF-1(PAO1-*LuxR*) | 0.63 |
| KF-1(PAO1) | 0.06 |
| KF-1(PAO1-*Sul1*) | 0.08 |

**Table S5** Strains and plasmids used in this study

| **Strain or plasmid** | **Relevant characteristics** |
| --- | --- |
| ***Comamonas testosteroni*** | |
| KF-1 | Wild-type |
| CNB-2/Δ*LuxR* | *LuxR* disrupted in CNB-2, Gm^R^ |
| ***Pseudomonas aeruginosa*** | |
| PAO1 | Wild-type |
| PAO1*-LuxR* |  |
| PAO1-*Sul1* |  |
| ***Escherichia coli*** | |
| BL21(DE3) | Expression host: F^-^ ompT r^-^_B_ m^-^_B_; DE3 is a λ derivative carrying lacI and T7 RNA polymerase genes under placUV5 control |
| pJQ200SK | Helper plasmid, Gm^R^ |
| pCVD442 | Suicide vector, Gm^R^ |
| pCVD442-Δ*LuxR*::Gm | Vector pCVD442 containing Gm gene cassette-truncated *LuxR* gene with flanking sequences for generating mutant Δ*LuxR* |
| pBBR1MCS2 | Broad host range of cloning vector, Km^R^ |
| pBBR1MCS2-*LuxR* | Complementation plasmid |
| pBBR1MCS2-*Sul1* | Complementation plasmid |

**Table S6** Primer pairs used in this study

| **Primers** | | **Description** | |
| --- | --- | --- | --- |
| **Used in construction of mutants lacking *LuxR*** | | | |
| *LuxR*-5F | CTACTCGCTCAGCCAGTTCACGTC, 5′ LuxR homologous arm | |  |
| *LuxR*-5R | GATTGCGATGCTCATCTCAGGCC | |  |
| *LuxR*-3F | GAACGCACTGTGGAAAACCACCTG, 3′ LuxR homologous arm | |  |
| *LuxR*-3R | CAAGATTCCGCACAGCCTGTTTGC | |  |
| *LuxR*-GmF | GGCCTGAGATGAGCATCGCAATCagaaatgcctcgacttcgc, primer for Gm^R^ gene amplification | |  |
| *LuxR*-GmF | CAGGTGGTTTTCCACAGTGCGTTCttaggtggcggtacttggg | |  |
| *LuxR*-outF | GCTGAACCATCGACTCCCGACAAGCAAC, external primer for mutant confirmation | |  |
| *LuxR*-outR | CTCGGCGTTACTCGCCAGCCTCTAC | |  |
| *LuxR*-inF | CTTCCAGCGCTCGAGACGATGCG, internal primer for mutant confirmation | |  |
| *LuxR*-inR | CGAATACAGGCACTTGCAGACCGTGG | |  |
| **Used in construction of complementation mutants carrying *LuxR* and *Sul1*** | | | |
| 415-F | caacgcaattaatgtgagttagctcac | |  |
| 415-R | ctcttcgctattacgccagctg | |  |

**Table S7** Selective agar media used in this study

| **Species type** | **Species** | **Selective plate** | **Incubation temperature (°C)** | **Time of colony appearance (hours)** | **Colony morphology** |
| --- | --- | --- | --- | --- | --- |
| **Cooperator** | *Comamonas testosteroni* KF-1 | NBA (Nutrient Broth agar, 4 g/100 mL) + sulfamethoxazole (14.25 μg/mL) | 30 | 24 | White colonies |
| **Cheater** | *Comamonas testosteroni* CNB-2/Δ*LuxR* | NBA (Nutrient Broth agar, 4 g/100 mL) + gentamycin (25 μg/mL) | 30 | 24 | White colonies |
| **Regulator** | *Pseudomonas aeruginosa* PAO1 | CNA (4 g/100 mL) | 30 | 24 | Green colonies |
|  | *Pseudomonas aeruginosa* PAO1-*LuxR* | CNA (4 g/100 mL)  + tetracycline (10 μg/mL) | 30 | 24 | Green colonies |
|  | *Pseudomonas aeruginosa* PAO1-*Sul1* | CNA (4 g/100 mL)  + tetracycline (10 μg/mL) | 30 | 24 | Green colonies |
